# Enhancing the Psychometric Properties of the Iowa Gambling Task Using Full Generative Modeling

**DOI:** 10.5334/cpsy.89

**Published:** 2022-08-26

**Authors:** Holly Sullivan-Toole, Nathaniel Haines, Kristina Dale, Thomas M. Olino

**Affiliations:** 1 Temple University, Department of Psychology and Neuroscience, US; 2 The Ohio State University, Department of Psychology, US

**Keywords:** Iowa Gambling Task, psychometrics, reinforcement learning, test-retest reliability, hierarchical Bayesian analysis, internalizing

## Abstract

Poor psychometrics, particularly low test-retest reliability, pose a major challenge for using behavioral tasks in individual differences research. Here, we demonstrate that full generative modeling of the Iowa Gambling Task (IGT) substantially improves test-retest reliability and may also enhance the IGT’s validity for use in characterizing internalizing pathology, compared to the traditional analytic approach. IGT data (n =50) was collected across two sessions, one month apart. Our full generative model incorporated (1) the Outcome Representation Learning (ORL) computational model at the person-level and (2) a group-level model that explicitly modeled test-retest reliability, along with other group-level effects. Compared to the traditional ‘*summary score*’ (proportion good decks selected), the ORL model provides a theoretically rich set of performance metrics (*Reward Learning Rate* (*A*+), *Punishment Learning Rate* (*A*-), *Win Frequency Sensitivity* (β*f*), *Perseveration Tendency* (β*p*), *Memory Decay* (*K*)), capturing distinct psychological processes. While test-retest reliability for the traditional summary score was only moderate (*r* = .37, BCa 95% CI [.04, .63]), test-retest reliabilities for ORL performance metrics produced by the full generative model were substantially improved, with test-retest correlations ranging between *r* = .64–.82. Further, while summary scores showed no substantial associations with internalizing symptoms, ORL parameters were significantly associated with internalizing symptoms. Specifically, *Punishment Learning Rate* was associated with higher self-reported depression and *Perseveration Tendency* was associated with lower self-reported anhedonia. Generative modeling offers promise for advancing individual differences research using the IGT, and behavioral tasks more generally, through enhancing task psychometrics.

## Introduction

Behavioral tasks offer the promise of objective assessment and probing psychological processes inaccessible via self-report; however, poor psychometrics are a barrier to using behavioral tasks for individual differences research (Cooper et al., 2017; Hedge et al., 2018; Rouder & Haaf, 2019). The Iowa Gambling Task (IGT; Bechara, 2007) is thought to measure real-world decision-making ability in the context of reward and risk. The IGT’s utility, however, is limited by inadequate reliability and validity (Buelow & Suhr, 2009; Schmitz, et al., 2020), likely stemming from the diversity of psychological processes driving task performance (Ahn, et al., 2008; Buelow & Suhr, 2013; Lin, et al., 2007; 2013; Worthy, Pang & Byrne, 2013) and poor measurement precision from traditional scoring. To address these issues with the IGT we used a generative model, broadly defined as a model that can generate (simulate) data consistent with the data being analyzed. More specifically, we demonstrate how generative modeling (Haines, et al., 2020)— a statistical modeling approach that better aligns model specification with theory while also accounting for measurement uncertainty across multiple levels—can be used to improve IGT test-retest reliability and may also elucidate links between distinct psychological processes driving IGT performance and internalizing symptomology.

### Hierarchical modeling to improve behavioral task psychometrics

Behavioral tasks are routinely used for individual differences research (e.g., correlating a task metric with a self-report trait measure); however, the psychometric properties of tasks have largely been neglected in psychological research (Parsons, Kruijt & Fox, 2019). Established behavioral tasks produce robust group-level effects through low between-person variance in task scores; however, low between-person variance results in low reliability for individual scores, which reduces task utility for individual difference inference (Cooper et al., 2017; Hedge et al., 2018). Recently, multiple independent groups have shown that hierarchical models can be used to better account for uncertainty in person-level model parameters, leading to increased reliability of measures derived from behavioral tasks (Brown, et al., 2020; Haines, et al., 2020; Rouder & Haaf, 2019).

### Psychometric issues arising from traditional analyses of the IGT

In the IGT, participants make selections among four decks of cards with different win/loss contingencies: two advantageous decks that net positive outcomes and two disadvantageous decks that net negative outcomes. Greater selection from disadvantageous decks is thought to reflect impaired value-based decision making and is associated with multiple forms of psychopathology (Mukherjee & Kable, 2014). Despite robust group-level associations between IGT performance and psychopathology, the standard analytical approach for the IGT leads to psychometric issues.

Analyses involving behavioral tasks can be conceptualized at two levels: (1) the person level, involving a behavioral model capturing individual task performance, and (2) the group level, involving a model that considers variation across people and is used for inference about broader-level phenomena (e.g., behavioral estimate’s change over time). Like many behavioral tasks, the IGT is typically analyzed using (1) a ‘*summary statistic*’ at the person-level (e.g., proportion of trials where person selects advantageous decks; we term such metrics ‘*IGT summary scores*’) and (2) a ‘*two-step approach*’ at the group level (e.g., taking the person-specific summary scores and using them as observed quantities in subsequent analyses (e.g., a test-retest correlation); we term this ‘*two-step summary approach*’). While the two-step summary approach is widely used with the IGT, it can contribute to poor construct validity (Buelow & Suhr, 2009) and poor test-retest reliability (Schmitz, et al., 2020).

#### IGT validity issues

With IGT summary scores, questions arise about what *exactly* the IGT is measuring. For example, as cards from the IGT involve combinations of monetary gains and losses, both approach and avoidance can drive performance (Cauffman, et al., 2010; Peters & Slovic, 2000), but these processes are not dissociable with a single summary score. Further, while many studies have reported differences in average summary scores between clinical groups and controls (see Mukherjee & Kable, 2014 for meta-analysis), the use of summary scores does not facilitate inference on *why* groups show performance differences. Additionally, IGT summary scores show inconsistent associations with criterion constructs (Buelow & Suhr, 2009). However, one relatively consistent pattern is that *both* state-level negative mood and trait-level approach motivation are associated with greater disadvantageous choice (Buelow & Suhr, 2013; Case & Olino, 2020; Suhr & Tsanadis, 2007; van Honk, et al., 2002), suggesting IGT summary scores do not represent one homogenous construct. Together, evidence suggests summarizing task behavior with a single metric confounds distinct processes contributing to observable behavior (Ahn, et al., 2016; Almy, et al., 2018; Cauffman, et al., 2010; Lin, et al., 2007).

#### IGT reliability issues

Studies have reported moderate-to-good same-day test-retest reliability for IGT summary scores (*r* = .57–.61, Lejuez et al., 2003; *r* = .36, Schmitz, et al., 2020). With intervals of 2–3 weeks, only moderate reliability has been reported (*r* = .27–.35; Buelow & Barnhart, 2018; Xu, et al., 2013). Such lower reliability hinders investigation of individual differences and change over time as it is impossible to dissociate variation in behavioral metrics arising from measurement uncertainty from variation in true effects of interest. The two-step summary approach ignores measurement uncertainty, which can attenuate subsequent correlations, thus compromising reliability and validity (Haines et al., 2020; Ly et al., 2017; Rouder & Haaf, 2019; Turner et al., 2017).

### Tackling psychometric issues with full generative models

*A full generative model* for behavioral task data combines within a single statistical model (1) a person-level model that specifies how observable behavior is generated by each person trial-by-trial as they engage with the task, and (2) a group-level model that captures variation in the behavioral model parameters across people (or other individual or group effects of interest). By estimating parameters from both levels of analysis simultaneously, the full generative approach takes into account multiple relevant sources of measurement uncertainty and produces better estimates of both person- and group- level parameters, thus enhancing the psychometrics of task-derived measures (Haines, et al., 2020).

#### Generative modeling at the person-level

At the person-level, generative modeling involves constructing a model that can simulate observable task behavior that is consistent with real data. Where traditional analysis involves person-level summary scores, our generative approach uses the Outcome-Representation Learning (ORL) computational model, a reinforcement learning (RL) model developed to dissociate different decision-making mechanisms driving IGT behavior (Haines et al., 2018). The ORL builds on previous computational models (Ahn, et al., 2008; Busemeyer & Stout, 2002; Worthy, Pang & Byrne, 2013) and has demonstrated good performance in predicting participants’ earnings and trial-to-trial choices (Haines, et al., 2018). The ORL assumes each person’s trial-level choices are governed by five parameters including: *Reward Learning Rate, Punishment Learning Rate, Win Frequency Sensitivity, Perseveration Tendency*, and *Memory Decay*, yielding person-level estimates for each. Thus, the ORL provides a theoretically rich set of measures capturing distinct psychological processes (see Table 1). Computational models have revealed meaningful differences in latent psychological processes between groups that are not observable with summary scores alone (Ahn, et al., 2014; Ahn, et al., 2016; Haines, Vassileva & Ahn, 2018; Kildahl, et al., 2020; Romeu, et al., 2020; Yechiam, et al., 2005). However, the ORL model itself may not be sufficient to remedy poor test-retest reliability, as parameters from other RL models, when modeled separately across time points, generally show only modest reliability (e.g., *r* = .48–.57, Chung, et al., 2017; *r* = .30, Moutoussis, et al., 2018; *r* = .63, Price, Brown & Siegle, 2019; *r* = .16–.20, Shahar, et al., 2019).

**Table 1:** **ORL Model and Parameter Computation.**


ORL MODEL PARAMETER	PARAMETER REPRESENTS	HIGHER VALUES INDICATE	EQUATION		COMPUTATION NOTES

***A +***	Reward/ Punishment Learning Rates	The rate at which an individual updates expected value and expected outcome frequency for a given deck following gains or losses, respectively	faster learning/ more volatile updating in a gains or loss domain, respectively	\[E{V_j}(t + 1) = \left\{ {\begin{array}{*{20}{l}} {E{V_j}(t) + {A_{{\rm {rew}}}} \cdot (x(t) - E{V_j}({\mathrm{t}})),}&{{\mathrm{if}}\;x(t) \ge 0}\\ {E{V_j}(t) + {A_{{\rm {pun}}}} \cdot (x(t) - E{V_j}({\mathrm{t}})),}&{{\mathrm{otherwise}}} \end{array}} \right.\]	Reward and punishment learning rates are estimated seperately and are shared between the *EV* computation (left) and the computation (below). Expected value is updated using objective outcome amount *x(t)*.

***A-***					

***βf***	Win Frequency Sensitivity	The effect of gain frequency (as opposed to outcome magnitude) on the subjective value for a given deck	greater preference for decks with a higher win frequency over objectively equivalent decks that win less often	\[E{F_j}(t + 1) = \left\{ {\begin{array}{*{20}{l}} {E{F_j}(t) + {A_{{\mathrm{rew}}}} \cdot (sgn(x(t)) - E{F_j}({\mathrm{t}})),}&{{\mathrm{if}}\;x(t) \ge 0}\\ {E{F_j}(t) + {A_{{\mathrm{pun}}}} \cdot (sgn(x(t)) - E{F_j}({\mathrm{t}})),}&{{\mathrm{otherwise}}} \end{array}} \right.\] \[E{F_{j\prime }}(t + 1) = \left\{ {\begin{array}{*{20}{l}} {E{F_j}\prime (t) + {A_{{\mathrm{pun}}}} \cdot \left({\frac{{ - sgn(x(t))}}{c} - E{F_j}\prime ({\mathrm{t}})} \right),}&{{\mathrm{if}}\;x(t) \ge 0}\\ {E{F_j}\prime (t) + {A_{{\mathrm{rew}}}} \cdot \left({\frac{{ - sgn(x(t))}}{c} - E{F_j}\prime ({\mathrm{t}})} \right),}&{{\mathrm{otherwise}}} \end{array}} \right.\]	Expected win frequency is tracked seperately from EV. The signum function (*sgn*(*x*(*t*)) returns 1, 0, or -1 for positive, 0, or negative outcome values on trial (*t*), respectively. Expected win frequency is also updated for unchosen decks (*j*’) on trial (*t*), where *C* is the number of possible alternative choices for the chosen deck (*j*) (here, 3).

***βp***	Perseveration Tendency	The tendency to stick with a previous selection (as opposed to switching among decks), regardless of outcomes	more choice consistency, less switching	\[P{S_j}(t + 1) = \left\{ {\begin{array}{*{20}{l}} {\frac{1}{{1 + K}}{\mathrm{ }},}&{{\mathrm{if}}\;D(t) = j}\\ {\frac{{P{S_j}(t)}}{{1 + K}}{\mathrm{ ,}}}&{{\mathrm{otherwise}}} \end{array}} \right.\]	The perseverance weight of the chosen deck (*j*) is set to 1 on each trial (*t*), and then the perseverance weights decay exponentially before a choice is made on the next trial.

***K***	Memory Decay	The extent to which an individual forgets their own history of selecting decks	greater forgetting; remembering a shorter (rather than longer) sequence of deck selections	\[K = {3^{K^{\prime}}} - 1\]	*K* is a decay parameter controlling how quickly decision makers forget past deck selections.


The ORL model assumes expected value *(EV)*, expected frequency *(EF)*, and choice perseverance *(PS)* signals are integrated linearly to generate a value signal for each deck (*j*) at time (*t*) as follows: \[{V_j}(t + 1) = E{V_j}(t + 1) + E{F_j}(t + 1) \cdot {\beta _f} + P{S_j}(t + 1) \cdot {\beta _P}\] To generate choice probabilities, the estimated value above is entered into a softmax function, where *D(t)* is the chosen deck at trial *t* as follows: \[{\mathrm{Pr}}[D(t + 1) = j] = \frac{{{e^{{V_j}(t + 1)}}}}{{\mathop \sum \nolimits_{K = {1^e}^{{V_k}(t + 1)}}^4 }}\] The five free parameters are computed as follows:

#### Generative modeling at the group-level

Unless person-level summary scores have perfect reliability (i.e. are estimated with perfect precision), any correlation obtained using the two-step approach will be attenuated toward 0—including test-retest and correlations with external measures (e.g., self-reports) (Spearman, 1904). If we are interested in the “true” test-retest correlation across sessions (i.e., construct stability across time, irrespective of the measure’s reliability within each session), we can use a hierarchical model to jointly estimate performance metrics across two sessions. Along with estimating person-level behavioral parameters, the hierarchical model simultaneously estimates test-retest correlations and other group-level effects. This joint estimation allows for information to be shared across people, inducing pooling of person-level parameters toward group-level means. When covariance across timepoints is included in the hierarchical model, within-person information is shared across timepoints, inducing pooling of person-level estimates toward a common person-level mean. This within-person pooling has the effect of disattenuating test-retest correlations for unreliability (e.g., Haines, et al., 2020; Brown, et al., 2020). Although joint generative modeling across multiple sessions of data has shown promise for enhancing psychometrics in other paradigms (Brown, et al., 2020), to date, this approach has not been taken to investigate the IGT’s test-retest reliability.

### Enhancing the IGT’s relevance for characterizing internalizing pathology

By improving psychometrics, full generative modeling may broaden the IGT’s utility to have better application to internalizing pathology. While the IGT has been widely used to study externalizing disorders (Mukherjee & Kable, 2014), less work has examined IGT performance in relation to internalizing. When IGT summary scores have been applied to internalizing, results are mixed (Paulus & Yu, 2012), with depression/anxiety symptoms linked to *both* impaired (Alacreu-Crespo, et al., 2020; Cella, et al., 2010; Miu, Heilman, & Houser, 2008; Moniz, et al., 2016; Must, et al., 2006; Rinaldi, et al., 2020) and enhanced (Byrne et al., 2016; Mueller, et al., 2010; Smoski, et al., 2008) performance, or no association between internalizing and performance (Baeza-Velasco, et al., 2020; Case & Olino, 2020; Jollant, et al., 2016; McGovern et al., 2014). Such inconsistencies may reflect the heterogeneity of processes driving IGT performance, obscured by summary scores, as well as measurement uncertainty.

Given strong theoretical relevance of RL for internalizing disorders (Bishop & Gagne, 2018; Chen, et al., 2015), computational models for the IGT have great potential for elucidating specific decision-making patterns within internalizing pathology, as already shown with substance abuse (Ahn, et al., 2014; Ahn, et al., 2016; Haines, Vassileva & Ahn, 2018; Kildahl, et al., 2020; Romeu, et al., 2020). RL models have revealed distinct phenomena related to initernalizing using the IGT (Alacreu-Crespo, et al., 2020; Byrne, et al., 2016; see Supplemental Background for details) and other learning tasks (Brown, et al., 2021; Chen, et al., 2015; Pizzagalli, et al., 2020); however, the strong-performing ORL model (Haines, et al., 2020) has not yet been used to examine internalizing despite its parameterization of highly relevant decision mechanisms. For example, the ORL calculates separate gain and loss learning rates, in line with neurobiological and behavioral evidence for dissociable reward and punishment learning systems (Christakou, et al., 2013; Frank, Seeberger & O’Reilly, 2004; Gershman, 2015). Separate parameterization may be relevant for characterizing the putative hypoactive reward system in depression and hyperactive punishment system in depression and anxiety (Bishop & Gagne, 2018; Clark & Watson, 1991). Further, the ORL includes a parameter for perseveration/choice consistency, deficits in which are implicated in depression and suicidality (Alacreu-Crespo, et al., 2020; Dombrovski & Hallquist, 2021).

### Goals of the current study

#### Modeling overview

**Figure 1:**
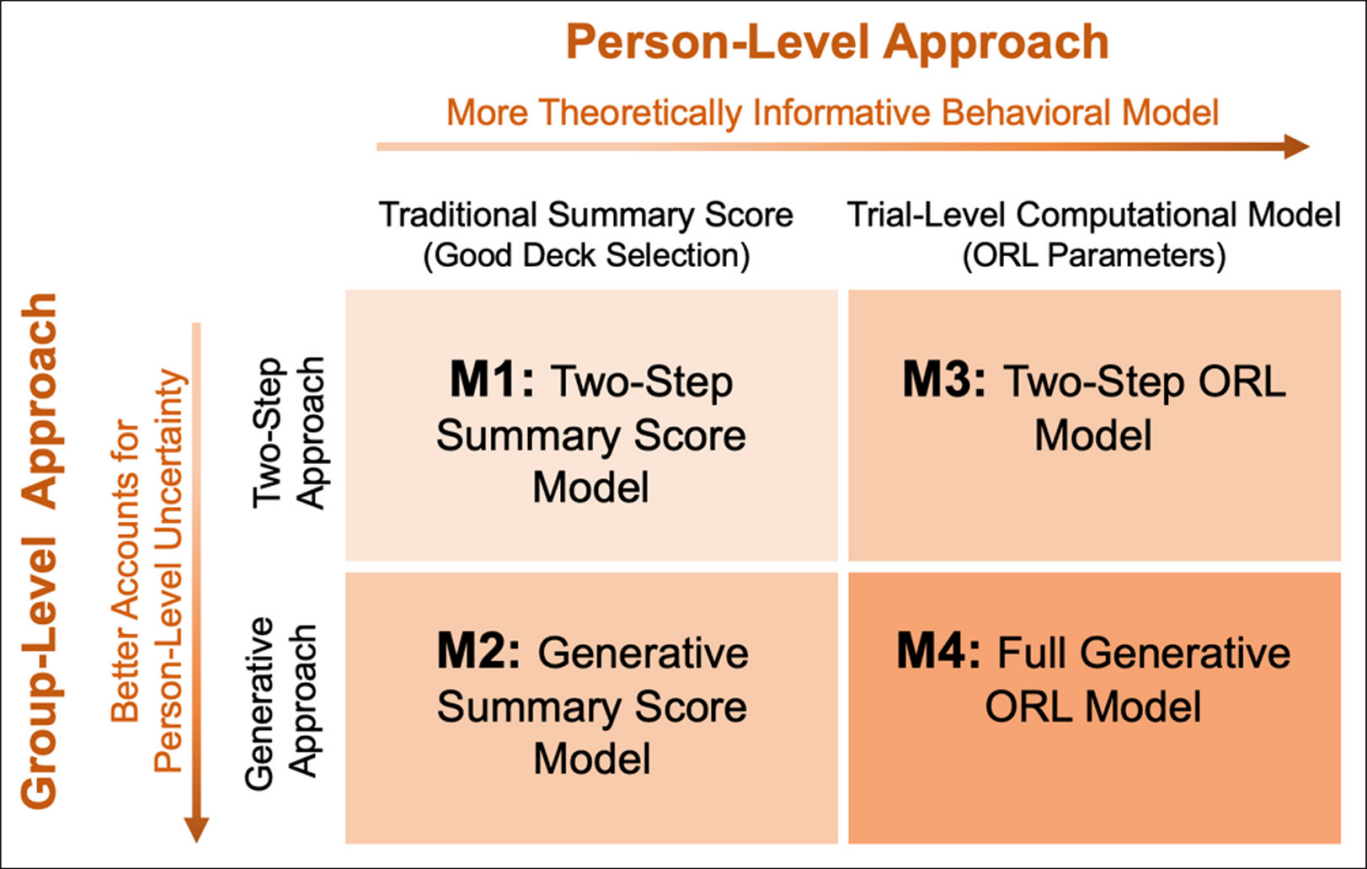
**Overall Modeling Approach and Resulting Four Models.** At the person-level, Models 1 and 2 used the traditional summary score (*proportion good deck selected*) to model gross task behavior and Models 3 and 4 used the ORL computational model to estimate trial-level task behavior in terms of five parameters (*Reward Learning Rate* (*A*+), *Punishment Learning Rate* (*A*-), *Win Frequency Sensitivity* (β*f*), *Perseveration Tendency* (β*p*), *Memory Decay* (*K*)). At the group-level, Models 1 and 3 estimated person-level metrics separately at each testing session and subsequently used these estimates in two-step test-retest correlations, and Models 2 and 4 used a generative approach to model person-level metrics (summary score or ORL parameters, respectively) across both testing sessions while simultaneously estimating, within the same hierarchical model, the test-retest associations between the model’s person-level metrics.

The current study examined whether full generative modeling could improve the psychometrics of IGT indices compared to the traditional two-step summary approach. Across four models, we evaluated how different modeling assumptions affected reliability and validity. More specifically, two person-level modeling approaches (summary score vs. ORL computational model) were “crossed” against two group-level modeling approaches (two-step approach vs. generative modeling across two testing sessions) to create four models of increasing complexity (see Figure 1). Model 1 relies on the two-step summary approach conventionally applied in IGT studies. Model 2 estimates a generative version of Model 1 that jointly estimates the person-level summary score (probability of choosing good versus bad decks) across both testing sessions while simultaneously estimating the test-retest correlation. Thus, Model 2 accounts for uncertainty in person-level estimates that Model 1 ignores but estimates a person-level metric analogous to that of Model 1. Model 3 estimates the person-level ORL parameters independently within each session and then estimates the test-retest correlation for each model parameter using a two-step approach. Model 4 estimates the person-level ORL parameters jointly across both sessions while simultaneously estimating the test-retest correlations for each parameter. Thus, Model 4 estimates the same person-level metrics (ORL parameters) as Model 3 but accounts for uncertainty in the person-level estimates.

#### Research questions

Our overarching hypothesis was that *both* the use of (1) a more theoretically informative person-level model (i.e., going from Model 1 to Model 3, and from Model 2 to Model 4) *and* the use of (2) hierarchical generative models that simultaneously estimate person-level parameters and their test-retest correlations (i.e. going from Model 1 to Model 2, and from Model 3 to Model 4) would yield behavioral estimates with increased utility for individual differences research. More specifically, we predicted that behavioral estimates from Model 4 would have the highest test-retest reliability. Additionally, as the ORL model provides more theoretically rich metrics and incorporates more behavioral information across time, we had a general prediction that the Model 4 estimates would show improved construct validity in relation to (a) an *a priori* set of trait and state self-report measures commonly associated with IGT performance as well as with (b) an *a priori* set of internalizing symptom measures that have not shown consistent associations with IGT performance across previous research; however, examining associations with self-report measures was exploratory with no specific hypotheses. As an undergraduate convenience sample was used, clinical implications are tentative.

## Method

### Participants and procedure

Data collection procedures were approved by the Temple University IRB (ref 22065), and participants provided written informed consent in accordance with the Declaration of Helsinki. The sample included 50 undergraduates (74% Female) recruited through SONA, an online research management platform, and received course credit for participation. Participant ages ranged from 18 to 24 years (mean = 19.98 years, *SD* = 1.6 years). The racial breakdown of our sample was 38% White (n = 19), 22% Black/African American (n = 11), 16% Asian (n = 8), 20% reporting Biracial or ‘Other’ (n = 10), and the sample was primarily non-Hispanic (74%). IGT behavioral data and self-report measures were collected across two testing sessions approximately one month apart. The full sample of 50 participants completed the IGT at session 1 and 46 participants completed the IGT at session 2. The generative models that simultaneously modeled both sessions (Models 2 and 4), estimated behavioral parameters at session 2 for the four missing participants, recovering the full sample. Self-report measures were available for 46 or 48 participants at session 1 and for 48 participants at session 2.

### Iowa Gambling Task (IGT)

The original version of the IGT (Bechara et al., 1994) was administered via E-Prime Stimulus Presentation Software (Schneider, Eschman & Zuccolotto, 2002). Participants are given a “bank” of $2000 and across 100 trials, participants are presented with four card decks from which they can draw a card and they freely choose among the decks. Cards yield a monetary gain and (sometimes) a monetary loss. Each deck has a different payout distribution, which, unbeknownst to the participant, is fixed such that the sequence of cards from any given deck is the same across participants (see Supplemental Table 1 for exact win/loss contingencies). There are two advantageous decks and two disadvantageous decks, with the advantageous decks initially yielding smaller gains and the disadvantageous decks initially yielding larger gains. All decks allow unlimited draws, and across 10 draws from each deck, Decks A and B (disadvantageous decks) have an average expected value of -$250, while Decks C and D (advantageous decks) have an average expected value of $250; however, the net value is not easy to calculate from the specific gain and loss values, obscuring the payout structure of the individual decks from explicit awareness. Thus, participants learn about the rewarding/punishing nature of the decks through sampling across 100 trials. Performance is traditionally measured in terms of the number of good decks selected. Participants were told that their game earnings would be exchanged for a real cash bonus; however, the exchange rate was not specified.

### Overview of modeling approaches

We “crossed” two person-level modeling approaches (summary score vs. ORL computational model) against two group-level modeling approaches (two-step approach vs. generative modeling across both timepoints) to create four models (see Figure 1), each yielding person-level behavioral metrics for each of the two sessions. Models 2, 3 and 4 yielded posterior distributions and posterior means for behavioral estimates. As mentioned, the generative models (Models 2 and 4) estimated parameters for the four subjects missing data at session 2, with the recovered missing data informed by both group-level effects and the given person’s own session 1 performance.

#### Overview of person-level models

##### Summary score

Model 1 computed the observed percentage of selections from a good deck for each participant at each session (traditional summary score). Model 2 used a binomial model to estimate ‘*theta*’ (*θ*), or the probability of selecting a good deck, a measure analogous to the traditional summary score, for each participant at each session.

##### Outcome-Representation Learning (ORL) computational model

Models 3 and 4 used the Outcome-Representation Learning (ORL) computational model, developed by Haines and colleagues, to model trial level choice behavior on the IGT. Compared to competing computational models for the IGT, the ORL has demonstrated better or commensurate performance across several metrics including post hoc model fit, simulation performance, and parameter recovery (Haines, et al., 2018). The ORL model assumes deck selection is guided by a value function informed by expected value (EV), expected frequency (EF) or a sensitivity to win frequency (rather than outcome magnitude), and choice perseverance (PS; choice consistency) and these terms are integrated in a linear fashion (see Table 1). Within this value function, the model estimates five free parameters: Reward Learning Rate (*A*+), Punishment Learning Rate (*A*-), Win Frequency Sensitivity (β*f*), Perseveration Tendency (β*p*), and Memory Decay (*K*). Reward and punishment learning rates modify prediction error to inform expected value and expected frequency, and the memory decay parameter modifies the perseveration term. The beta parameters (β*f* and β*p*) capture the extent to which win frequency and past deck choice (i.e. perseveration) influence an individual’s current choice. If β*f* = β*p* = 0, then only expected value information is taken into account. Otherwise, positive or negative beta parameters indicate that win frequency/past deck choice increase or decrease the likelihood of choosing a given deck. For example, if β*p* is positive, then people are more likely to continue choosing the same deck. However, if β*p* is negative, people are more likely to switch. Note that the ORL does not contain an inverse temperature parameter. Overall, the ORL yields a theoretically rich set of measures that capture distinct psychological processes underlying performance on the IGT.

#### Overview of group-level approaches to modeling test-retest reliability

##### Two-step approach

Models 1 and 3 used a two-step approach for estimating test-retest associations for each model’s respective person-level behavioral metrics (summary score for Model 1; ORL parameter estimates for Model 3). Thus, for Model 1 and 3, estimating test-retest reliability entailed (1) estimating each model’s behavioral metrics separately for each session and (2) in a subsequent, independent analysis, estimating the correlation between the session 1 and session 2 behavioral estimates. This two-step approach ignores measurement uncertainty in the person-level behavioral estimates.

##### Generative approach

Models 2 and 4 took a generative approach using hierarchical Bayesian analysis (HBA) to estimate the respective person-level behavioral metrics (*θ* for Model 2; ORL parameters for Model 4) from *both* sessions *jointly* in a single model that simultaneously estimated group-level effects, including test-retest reliability. These hierarchical models pooled information across individuals and across sessions, regressing person-level estimates toward group-level means. Thus, unlike Models 1 and 3, the parameter estimates for Models 2 and 4 were informed by data from both sessions. Model 2 is generative in the sense that it can simulate data consistent with the original dataset (two sessions of individual summary scores). Model 4 is considered a ‘full generative model’ because it can (using the ORL model) generate trial-level choice data as if a participant was playing through the task (like Model 3) but can also generate two sessions of data for each participant, thus generating a full dataset. For estimating group-level effects in these generative models, model priors (detailed below and in the Supplement) assumed group-level distributions over person-level estimates. Model 2 and Model 4 assumed that person-level parameters followed multivariate normal distributions, which allowed us to estimate the test-retest correlation of parameters across sessions. Generative modeling that incorporates hierarchical estimates of test-retest reliability can improve the precision of behavioral estimates, thus, strengthening (or dis-attenuating) test-retest correlations and likely bolstering the utility of these model-derived metrics for individual difference inferences (Haines et al, 2020).

### Assessing reliability and validity

#### Test-retest reliability

For Models 1 and 3, test-retest reliability was assessed using a two-step approach in which behavioral estimates from each session were correlated in a separate subsequent analysis, and 95% bias-corrected and accelerated bootstrapped confidence intervals (95% BCa CIs; discussed below) were calculated. Models 2 and 4 (the generative models) estimated test-retest reliabilities for the respective model metrics directly within the hierarchical model, yielding a posterior distribution (and a posterior mean) for each of the respective reliability coefficients, and 95% credible intervals (95% CI; discussed below) were calculated. The method by which two-step estimates of test-retest (Models 1 and 3) were compared to their respective generative model estimates of test-retest (Models 2 and 4) is discussed further in the section ‘Credible intervals (95% CI) and HDI plots for HBA estimates’

#### Construct validity

To examine construct validity, we used two-step correlations between behavioral estimates and self-report scores on an *a priori* set of trait and state self-report measures commonly associated with IGT performance as well as with an *a priori* set of internalizing symptom measures. Two-step correlations were used to provide a fair comparison of construct validity across all models, and 95% BCa CIs were calculated. Specifically, self-report scores were correlated with the summary score for Model 1 and with posterior means estimated from Models 2–4. While we expected Model 4 estimates (that incorporated the most data) would exhibit the strongest construct validity, no specific or directional associations were hypothesized. As analyses estimating associations between HBA-derived behavioral estimates and self-report measures were exploratory, no hypotheses were specified at the level of individual correlations, and Null Hypothesis Significance Testing was not a goal; thus, no metrics related to statistical significance are reported. Further, regarding two-step correlations between HBA-derived estimates and self-report measures, data simulations have shown that two-step correlations including a point estimate (e.g., score from a self-report measure) and a posterior mean generated from a hierarchical model are generally conservative estimations of population correlations (Katahira, 2016). As a result, power to detect correlations between model parameters and external covariates is reduced. Therefore, 95% BCa CIs reported for correlations involving HBA-derived estimates and self-report measure should be interpreted with this limitation in mind.

#### Credible intervals (95% CI) and HDI plots for HBA estimates

To depict effects involving the HBA-derived estimates in a manner that illustrates uncertainty of model estimates, we used 95% Highest Density Interval (HDI) plots, implemented with the *hBayesDM* R toolbox (Ahn, Haines, & Zhang, 2017). A 95% HDI plot illustrates a full posterior probability distribution, with sample estimates plotted as a histogram and 95% credible intervals (95% CI), indicating that 95% of the estimates lie within the demarcated interval (here, a horizontal red line), where every estimate inside the interval is more probable than every estimate outside of the interval. HDI plots were used to illustrate both (1) performance differences between testing sessions for the generative model estimates (Supplemental Figure 3B and Supplemental Figure 4) and (2) test-retest estimates from the two-step versus generative models (Figure 3A and Figure 4A).

Two-step estimates of test-retest for Model 1 and Model 3 were compared to the generative model estimates of test-retest from Model 2 and Model 4, respectively. Where two-step estimates of test-retest reliability were simply Pearson’s r coefficients, the generative models produced model-generated estimates of test-retest reliability coefficients with full posterior distributions. HDI plots illustrated posterior distributions for the given generative reliability coefficients (from either Model 2 or Model 4), along with their posterior means. We also displayed (on these same HDI plots), the two-step estimates of test-retest reliability from the analogous model fitted separately at each time point (Model 1 or Model 3, respectively). The 95% HDI for the generative test-retest estimates were compared to the respective single values resulting from the two-step test-retest estimations to determine whether 95% HDI estimates overlapped with two-step estimates.

#### BCa bootstrapped confidence intervals (95% BCa CIs)

Bias-corrected and accelerated bootstrapped confidence intervals (95% BCa CIs) correct for bias and skewness in the distribution of bootstrap estimates and were implemented using the *wBoot* R package (Weiss, N. A., 2019). As discussed above, due to hierarchical model pooling of individual estimates toward group-level means (shrinkage), 95% BCa CIs reported for correlations involving HBA-derived estimates should be interpreted with some caution.

### Model summaries and further details

#### Model 2 details

Estimates of *θ* (the probability of selecting a good deck) at each session were assumed to be multiple normally distributed, such that person-level *θ*´s were drawn from group-level normal distributions for each session. In addition to the group-level normal distributions of *θ* for each session, the assumed multivariate normal distribution also included a covariance matrix constructed from a uniform distribution of standard deviations for *θ* and a correlation matrix (which provides the test-retest reliability coefficient). There was an LKJ(1) prior on the correlation matrix, which assumes a uniform distribution between -1 and 1, meaning that all possible values for the reliability coefficient were assumed to be equally likely. The model was sampled for 1,000 iterations, with the first 200 as warmup, across four sampling chains for a total of 3,200 posterior samples for each parameter.

#### Model 3 details

The ORL model is available within the easy-to-use *hBayesDM* R toolbox (Ahn, Haines, & Zhang, 2017) and is described in detail by Haines and colleagues (2018). The value function, softmax action selection policy, and individual parameter computations from the ORL are outlined here in Table 1. The five free parameters were estimated using HBA. Person-level parameters were assumed to be drawn from group-level distributions. Group-level distributions were assumed to be normally distributed, with priors for the group-level distributions’ means and standard deviations assigned normal distributions (or, for unbounded parameters, β*f* and β*p*, standard deviations were assigned to half-Cauchy distributions). See Haines and colleagues (2018) for further details of parameterization. The model was sampled for 1,500 iterations, with the first 500 as warmup, across four sampling chains for a total of 4,000 posterior samples for each parameter.

#### Model 4 details

Model 4 was the full generative model, incorporating all behavioral data (both sessions) in a single model. Like Model 3, the ORL computational model (Haines, Vassileva & Ahn, 2018) was used to estimate person-level task behavior using trial-level information, yielding a set of five parameter estimates (*A*+, *A*-, *K*, β*f*, and β*p*; see Table 1) for each session. Person-level parameters across sessions were assumed to follow from group-level multivariate normal distributions, where each separate parameter had its own multivariate normal distribution. Using the Reward Learning Rate *A*_+_ as an example:


\[\left[ {\begin{array}{*{20}{c}}
{{{\boldsymbol{\Phi}} ^{ - 1}}({A_ + }_{i,1})}\\
{{{\boldsymbol{\Phi}} ^{ - 1}}({A_ + }_{i,2})}
\end{array}} \right]{\sim} {\rm {MVNormal}}\left({\left[ {\begin{array}{*{20}{c}}
{{\mu _{{{\mathrm{A}}_ + },1}}}\\
{{\mu _{{{\mathrm{A}}_ + },2}}}
\end{array}} \right]{\mathrm{}},{\mathrm{}}{{\bf{S}}_{{A_ + }}}} \right)\]

Here, A+_*i*__,1_ and A+_*i*__,2_ are the Reward Learning Rates for person *i* at both session 1 and 2, respectively. *Φ*^–1^(…) is the inverse of the cumulative distribution function for the standard normal distribution, which is used because the person-level learning rates must fall between 0 and 1. Because *Φ*^–1^(…) transforms from [0,1] → [–∞, +∞], it allows for us to use the multivariate normal group-level distribution to capture the test-retest correlation despite the multivariate normal distribution itself having support outside of [0,1]. *µ*_A+,1_ and *µ*_A+,2_ are the group-level means for *A*_+_ at sessions 1 and 2, and ***S***_*A*__+_ is a covariance matrix that captures the correlation between the person-level parameters across sessions. Specifically, ***S***_*A*__+_ can be decomposed into the group-level standard deviations at each session (σ_A+,1_ and σ_A+,2_) and the 2 × 2 correlation matrix of interest, **R**_*A*__+_:


\[
{\bf{S}}_{A_{+}}= \left(\begin{array}{*{20}{l}} \sigma_{A_{+}, 1} & 0\\
0 & \sigma_{A_{+}, 2} \end{array}\right)
{\bf{R}}_{A_{+}}= \left(\begin{array}{*{20}{l}} \sigma_{A_{+}, 1} & 0\\
0 & \sigma_{A_{+}, 2} \end{array}\right)
\]

The off-diagonal of **R**_*A*__+_ contains one free parameter which indicates the test-retest correlation—the value that we present throughout the text. Finally, we assume the following LKJ prior on the correlation matrix:


\[{{\bf{R}}_{{A_ + }}} {\sim} \,\, {\rm {LKJcorr}}(1)\]

Because **R**_*A*__+_ contains only a free parameter, this prior equates to a uniform distribution between -1 and 1, meaning that all possible values for the test-retest correlation were assumed to be equally likely.

Priors for the group-level distributions’ means and standard deviations were assigned to normal distributions. For unbounded parameters, β*f* and β*p*, standard deviations were assigned to half-Cauchy distributions. We include details on the complete parameterization of models in the supplemental text. The model was sampled for 5,000 iterations, with the first 1,000 as warmup, across six sampling chains for a total of 24,000 posterior samples for each parameter.

#### HBA model implementation

HBA for Models 2, 3, and 4 were conducted using the Stan package version 2.16.0 (Carpenter, et al., 2016), a probabilistic programming language, which uses Hamiltonian Monte Carlo, a variant of the Markov chain Monte Carlo (MCMC) method, to sample from high-dimensional probabilistic models. The RStan package (Stan Development Team, 2017) was used to interface with Stan and all additional analyses were conducted in R. For all HBA analyses, convergence to target distributions was checked visually by observing trace-plots and numerically by computing *Rˆ* statistics for each parameter (Gelman & Rubin, 1992). *Rˆ* values for all models were below 1.1, suggesting that the variance between chains did not outweigh variance within chains. We used non-centered parameterizations of the group-level distributions in all hierarchical models to improve convergence and estimation efficiency. Model specifications are in the Supplemental Text. De-identified data and Model 4 code for results and figures are available on OSF: https://osf.io/b3kwz/?view_only=0cfa92d49a5e466cb55b2dc9a145f5ec.

#### Model validation

Model validation included both parameter recovery and posterior predictive checks. Results from posterior predicative checks (see Supplemental Figures 1 & 2) demonstrated that the simulated data was a good fit to the observed data. Parameter recovery in the current sample found that recovery statistics were acceptable across all parameters (see Supplement for details). Further, parameter recovery was conducted during the development of the ORL model, and Haines and colleagues (2018) found the ORL to have good recovery of both parameter means and of the full posteriors compared to competing models.

### Self-report measures

To assess general construct validity, we used trait- and state- level self-report measures commonly associated with IGT performance. The Behavioral Inhibition/Behavioral Activation Scales (BIS/BAS; Carver & White, 1994) measured trait-level sensitivities of the avoidance and approach systems. While the BIS scale is a unidimensional construct measuring sensitivity to negatively valenced events, the BAS scale has three subscales: BAS-Drive measures persistent pursuit of goals; BAS-Fun Seeking measures desire for and spontaneous approach toward new rewards; and BAS-Reward Responsiveness measures positive responses to the anticipation or consummation of rewards. State-level mood was assessed using the Positive Affect/Negative Affect Schedules (PANAS; Watson, Clark, & Tellegen, 1988), with participants rating same-day feelings for 10 items assessing positive emotion (PA subscale) and 10 items assessing negative emotion (NA subscale). BIS/BAS and PANAS were administered at session 1 (n = 48) and session 2 (n = 46).

To assess internalizing symptoms experienced in the past week, participants completed a 62-item version of the Mood and Anxiety Symptom Questionnaire (MASQ-Short Form; Clark & Watson 1991; Watson, et al., 1995). The MASQ has four subscales: MASQ-General Distress Anxiety and MASQ-General Distress Depression measure general anxious and depressed moods, respectively; MASQ-Anxious Arousal measures somatic hyperarousal; and MASQ-Anhedonic Depression measures low positive affect. The 14-item Snaith-Hamilton Pleasure Scale measured participants’ experience of pleasure in the last few days (SHAPS; Snaith et al., 1995). The Patient-Reported Outcomes Measurement Information System (PROMIS) Depression scale assessed past-week depression symptoms (PROMIS-Depression; 28-item; Cella, et al., 2007). MASQ and PROMIS-Depression were administered only at session 1 (n = 46) and SHAPS was administered at session1 (n = 48) and session 2 (n = 46). See Supplemental Tables 2 and 3 for self-report in-sample reliabilities and descriptive statistics.

## Results

### Performance across testing sessions

Using both ‘summary score models’, performance means were similar across sessions, while the standard deviation in performance was greater at session 2 (see Supplemental Figure 3). There was no strong evidence of group-level between-session differences for Model 3 parameters (see Supplemental Figure 4).

### ORL parameter associations with the summary score

**Figure 2:**
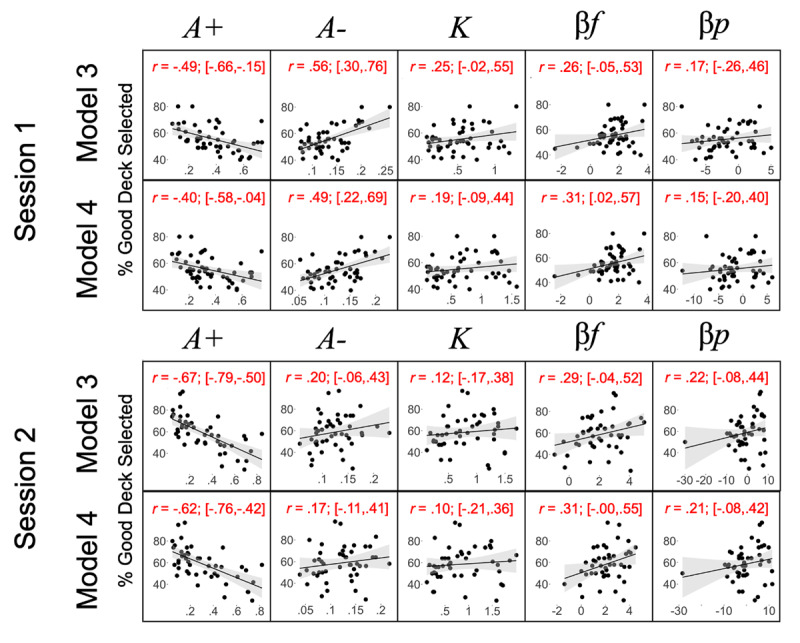
**Associations between ORL Parameters and the Summary Score.** Scatterplots represent the association between the Model 1 summary score, ‘percentage good deck selected’ (x-axis) and the posterior means for each of the ORL parameters (y-axis; Reward Learning Rate (*A*+), Punishment Learning Rate (*A*-), Win Frequency Sensitivity (β*f*), Perseveration Tendency (β*p*), and Memory Decay (*K*)), for Models 3 and 4, for each testing session. Interestingly, the influences of Reward and Punishment Learning Rates on overall performance appeared to be strengthened and attenuated, respectively, for session 2 compared to session 1.

The direction of associations between the summary score (a measure of overall good performance) and ORL parameter posterior means was consistent across the two sessions (see Figure 2). Reward Learning Rate (*A*+) was negatively associated with overall good performance, while all other parameters showed positive associations. Interestingly, both Reward and Punishment Learning Rates appeared to (oppositely) drive overall performance in session 1; while in session 2, their influences on overall performance appeared to be strengthened and attenuated, respectively. One outlier was identified at session 2 for the β*p* parameter; correlations excluding this participant were not substantially different (see Supplemental Table 5).

### Test-retest reliability across models

#### Reliability of the summary score: Model 1 versus Model 2

**Figure 3:**
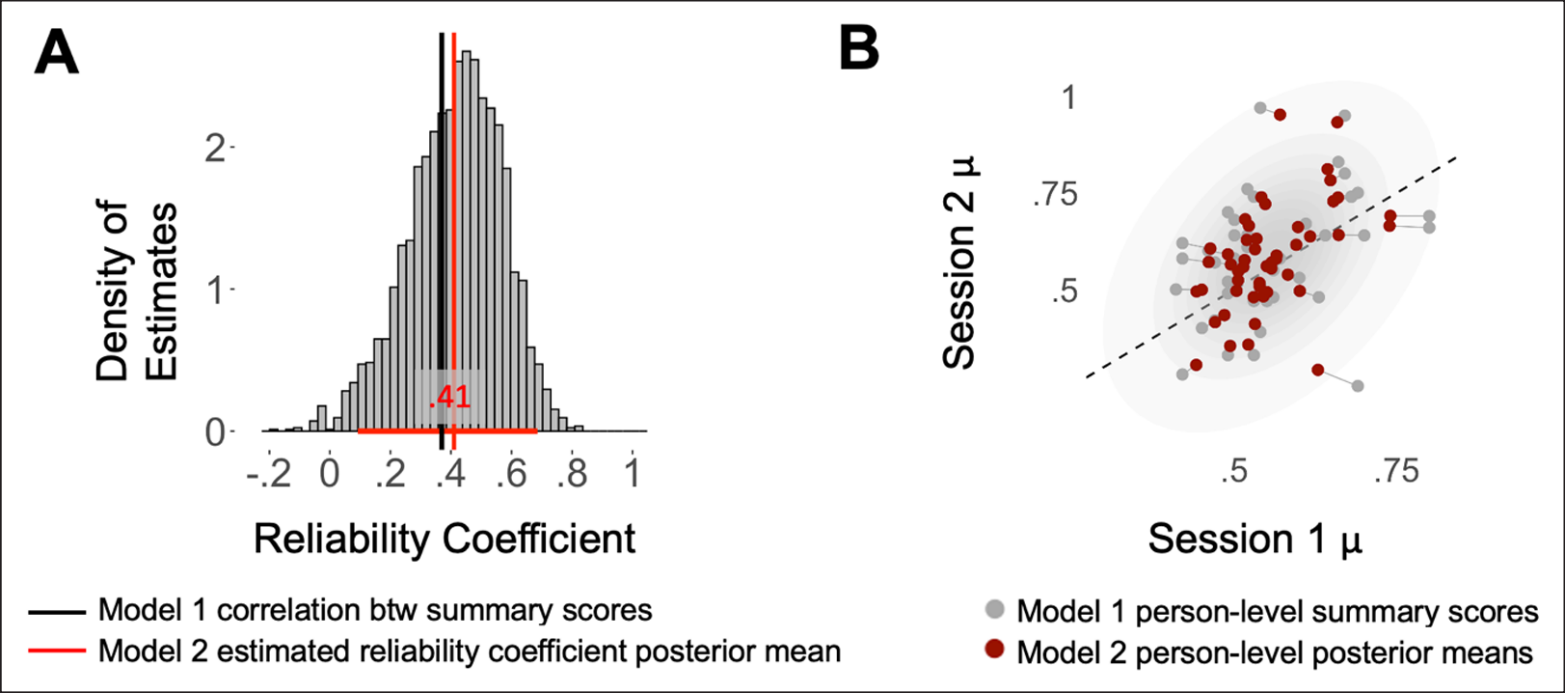
**Model 1 versus Model 2 Summary Scores and Test-Retest Reliability. (A)** HDI plot showing the posterior distribution of Model 2 estimated test-retest reliability coefficient for *θ*. The 95% highest density interval of estimates is indicated by the horizontal red line, and the vertical red line indicates the posterior mean for Model 2’s estimated test-retest reliability coefficient (*r* = .41). The Model 1 two-step test-retest reliability coefficient (Pearson’s *r*) for the summary score (*r* = .37) is indicated by the solid black line. **(B)** The relationship between the Model 1 and Model 2 estimates. Model 1 data points represent observed summary score means (‘percentage good deck selected’) at each of the two testing sessions (two-step approach). Model 2 data points represent the generatively modeled person-level posterior means for *θ* (‘probability of good deck selection’), modeled jointly across sessions. Grey lines connect Model 1 and Model 2 estimates for each participant, demonstrating the effect of the hierarchical model pooling estimates toward group-level means. The dashed grey line represents a perfect test-retest correlation of *r* = 1.

Test-retest reliability results for the Model 1 and Model 2 metrics are illustrated in Figure 3A. The test-rest reliability for the Model 1 (observed) summary score (*r* = .37, BCa 95% CI [.04, .63]) and for Model 2’s (jointly estimated) posterior mean for the reliability of *θ* (*r* = .41, 95% CI = [.09, .69]) were both only moderate with a wide 95% BCa confidence interval and 95% credible interval, respectively. Figure 3B shows the relationship between Model 1 and Model 2 estimates, demonstrating the effect of the hierarchical model pooling individual estimates toward group-level means. Despite hierarchical pooling, Model 2, which utilizes a simple person-level behavioral model (*θ*), did not substantially improve test-retest reliability compared to the traditional analytic approach. All r-values represent Pearson’s correlation between session 1 and session 2 metrics.

#### Reliability of the ORL parameters: Model 3 versus Model 4

**Figure 4:**
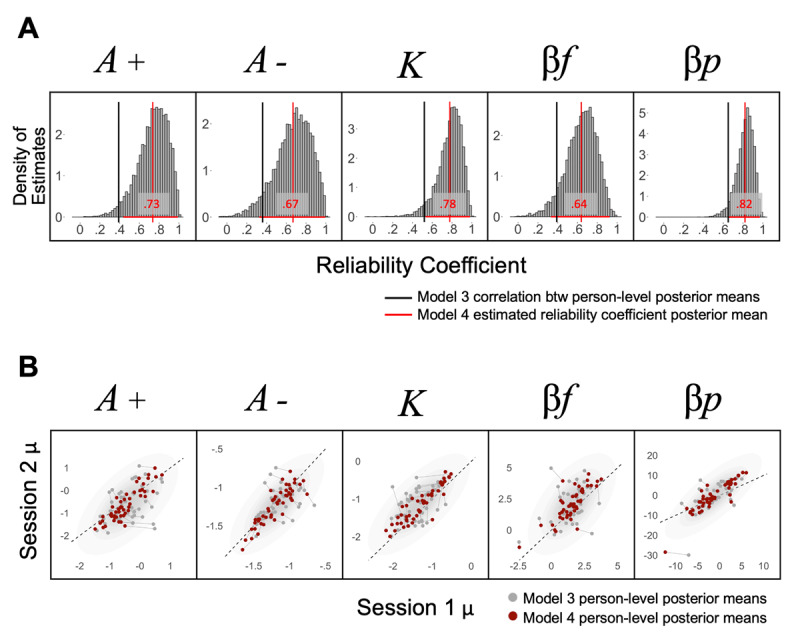
**Model 3 versus Model 4 Metrics and Test-Retest Reliability. (A)** HDI plots showing the posterior distributions of the Model 4 estimated test-retest reliability coefficients for each of the ORL five free parameters. The 95% highest density intervals for Model 4 estimates are indicated by horizontal red lines, and vertical red lines indicate posterior means for the Model 4 estimated test-retest reliability coefficients (*A*+ *r* = .73; *A*- *r* = .67; *K r* = .78; β*f r* = .64; β*p r* = .82). The Model 3 two-step test-retest reliability coefficients (Pearson’s *r*; *A*+ *r* = .39; *A*- *r* = .36; *K r* = .52; β*f r* = .39; β*p r* = .65) are indicated by solid black lines. **(B)** The relationship between the Model 3 and Model 4 estimates. Model 3 data points represent person-level posterior means for the ORL parameter estimates modeled separately at each of the two testing sessions. Model 4 data points represent generatively modeled person-level posterior means for the ORL parameter estimates, modeled jointly across testing sessions (full generative approach). Grey lines connect Model 3 and Model 4 estimates for each participant, demonstrating the effect of the hierarchical model pooling individual estimates toward group-level means. Dashed grey lines represent perfect test-retest correlation of *r* = 1.

Test-retest reliability for the Model 3 and Model 4 metrics (ORL five free parameters) is illustrated in Figure 4A. Test-retest reliability was moderate for the Model 3 two-step estimates (with 95% BCa confidence intervals: *A*+ *r* = .39, [.10, .61]; *A*- *r* = .36, [.05, .59]; *K r* = .52, [.25, .71]; β*f r* = .39, [–.02, .68]; β*p r* = .65, [.39, .76]), but test-retest reliability was substantially improved across all parameters for the Model 4 estimates (posterior means for reliability with 95% credible intervals: *A*+ *r* = .73, [.44, .99]; *A*- *r* = .67, [.33, .99]; *K r* = .78, [.53, .98]; β*f r* = .64, [.33, .92]; β*p r* = .82, [.65, .97]), with 95% CIs for the *A*+ and *K* parameters indicating that 95% of the estimate samples for reliability were stronger than (and completely non-overlapping with) the respective Model 3 two-step reliability estimates. Figure 4B compares the Model 3 and Model 4 estimates and demonstrates how full generative modeling improves test-retest reliability through the hierarchical model pooling individual estimates toward group-level means.

### Construct validity across models

#### Construct validity for Model 1 and Model 2

Associations between self-report measures and the Model 1 (observed) summary score and Model 2 (estimated) summary score are shown in Table 2. Across Models 1 and 2, summary scores showed moderate negative correlations with trait-level Behavioral Activation Drive (Model 1 *r* = –.38, BCa 95% CI [–.66, –.05]; Model 2 *r* = –.37, BCa 95% CI [–.65, –.03]) at the first session and showed moderate negative correlations with state-level Positive Affect (Model 1 *r* = –.30, BCa 95% CI [–.50, –.06]; Model 2 *r* = –.29, BCa 95% CI [–.49, –.06]) and state-level Negative Affect (Model 1 *r* = –.40, BCa 95% CI [–.62, –.13]; Model 2 *r* = –.40, BCa 95% CI [–.61, –.13]) at the second session. Thus, associations were not consistent across sessions. While the summary scores showed associations with state- and trait-level emotion and personality measures collected at the same session, associations with internalizing symptom measures from the same session were generally weak. See Supplemental Table 8 for associations between Session 1 self-report and Session 2 IGT metrics.

**Table 2:** **Model 1 and Model 2 Construct Validity.** Correlations between self-report measures and Model 1 and Model 2 summary scores. Correlations with 95% BCa CIs that do not include zero are bolded.


*SELF-REPORT COLLECTED AT SAME SESSION*	MODEL 1		MODEL 2	
	
	PERCENTAGE GOOD DECK SELECTED		PROBABILITY OF GOOD DECK SELECTION (θ)	
	
	SESSION 1	SESSION 2	SESSION 1	SESSION 2

BAS Total	–.25 [–.53, .05]	.09 [–.17, .36]	–.24 [–.52, .05]	.08 [–.18, .37]

BAS Drive	**–.38 [–.66, –.05]**	–.04 [–.34, .27]	**–.37 [–.66, –.03]**	–.05 [–.35, .26]

BAS Fun	–.10 [–.40, .20]	.13 [–.16, .40]	–.08 [–.38, .20]	.12 [–.15, .39]

BAS Reward Responsivity	–.14 [–.43, .13]	.13 [–.16, .39]	–.14 [–.42, .12]	.12 [–.17, .39]

BIS Total	–.20 [–.49, .09]	–.03 [–.34, .25]	–.19 [–.48, .12]	–.04 [–.33, .25]

PANAS PA	.02 [–.22, .25]	**–.30 [–.50, –.06]**	.01 [–.25, .24]	**–.29 [–.49, –.06]**

PANAS NA	–.13 [–.33, .07]	**–.40 [–.62, –.13]**	–.14 [–.34, .05]	**–.40 [–.61, –.13]**

MASQ General Distress Anxious	–.13 [–.39, .21]		–.14 [–.40, .21]	

MASQ Anxious Arousal	–.19 [–.45, .08]		–.21 [–.46, .07]	

MASQ General Distress Depressive	.01 [–.27, .39]		–.02 [–.29, .36]	

MASQ Anhedonic Depression	.11 [–.23, .46]		.11 [–.25, .47]	

SHAPS	.01 [–.24, .27]	.04 [–.21, .31]	.01 [–.24, .26]	.05 [–.21, .30]

PROMIS-D	.15 [–.16, .52]		.12 [–.20, .49]	


^a^ At session 1, the n for MASQ and PROMIS-D correlations is 46; the n for all other session 1 correlations is 48. ^b^ At session 2, the n for all correlations is 46.

#### Construct validity for Model 3 and Model 4

Associations between self-report measures and the Model 3 and Model 4 ORL parameters are shown in Table 3. Patterns of association were generally similar, with Model 4 estimates showing generally stronger associations with self-report. While Model 1 and 2 estimates were only weakly associated with internalizing symptoms, Model 4 ORL estimates showed some moderate correlations with internalizing symptoms.

**Table 3:** **Model 3 and Model 4 Construct Validity.** Correlations between self-report measures and ORL model estimates. Correlations with 95% BCa CIs that do not include zero are bolded.


MODEL 3										

ORL ESTIMATES (MODELED SEPARATELY AT EACH SESSION)										

*SELF-REPORT COLLECTED AT SAME SESSION*	SESSION 1					SESSION 2				
	
	*A+*	*A-*	*K*	*βf*	*βp*	*A+*	*A-*	*K*	*βf*	*βp*

BAS Total	.03 [–.29, .30]	–.17 [–.52, .17]	.02 [–.35, .33]	–.11 [–.42, .19]	.08 [–.25, .41]	.01 [–.26, .27]	.15 [–.11, .41]	.12 [–.17, .43]	–.03 [–.30, .23]	.24 [–.03, .49]

BAS Drive	.02 [–.34, .36]	–.29 [–.60, .07]	–.05 [–.39, .24]	–.07 [–.38, .20]	.01 [–.33, .38]	.08 [–.20, .37]	.10 [–.16, .37]	.10 [–.18, .38]	.00 [–.25, .24]	.21 [–.09, .48]

BAS Fun	.05 [–.23, .33]	–.01 [–.36, .28]	.09 [–.25, .36]	–.11 [–.37, .16]	.14 [–.16, .42]	.02 [–.23, .28]	.18 [–.10, .43]	.13 [–.20, .47]	.07 [–.24, .35]	.17 [–.13, .40]

BAS Reward Responsivity	.01 [–.26, .26]	–.10 [–.43, .18]	.02 [–.36, .33]	–.10 [–.40, .18]	.06 [–.26, .35]	–.09 [–.36, .16]	.08 [–.18, .33]	.06 [–.24, .33]	–.13 [–.41, .14]	.17[–.06, .40]

BIS Total	.02 [–.26, .29]	–.17 [–.47, .13]	–.32 [–.59, .08]	–.20 [–.51, .09]	–.18 [–.46, .20]	.00 [–.34, .31]	.08 [–.17, .30]	.02 [–.28, .33]	–.21 [–.49, .07]	–.01 [–.27, .25]

PANAS PA	–.08 [–.36, .18]	–.20 [–.44, .07]	.07 [–.24, .40]	–.11 [–.37, .20]	.14 [–.15, .42]	**.31 [.03, .50]**	.06 [–.25, .45]	–.18 [–.43, .11]	–.07 [–.29, .18]	.00 [–.33, .37]

PANAS NA	.09 [–.17, .31]	–.09 [–.31, .12]	–.04 [–.36, .34]	**–.29 [–.52, –.01]**	.08 [–.20, .37]	**.40 [.16, .60]**	.14 [–.15, .40]	–.05 [–.30, .27]	–.01 [–.37, .26]	.00 [–.24, .29]

MASQ General Distress Anxious	.07 [–.23, .34]	.10 [–.20, .36]	–.07 [–.35, .25]	–.16 [–.39, .14]	–.06 [–.35, .24]					

MASQ Anxious Arousal	.13 [–.14, .38]	.12 [–.12, .37]	–.06 [–.36, .31]	–.11 [–.37, .28]	–.11 [–.40, .19]					

MASQ General Distress Depressive	.05 [–.25, .31]	**.28 [.02, .60]**	.07 [–.26, .45]	–.06 [–.49, .27]	–.13 [–.43, .21]					

MASQ Anhedonic Depression	–.08 [–.39, .23]	.18 [–.15, .51]	.03 [–.29, .36]	.01 [–.33, .27]	–.05 [–.37, .27]					

SHAPS	–.09 [–.35, .20]	–.12 [–.35, .16]	.11 [–.21, .42]	–.10 [–.35, .16]	.27 [–.01, .51]	–.15 [–.41, .14]	–.04 [–.30, .19]	.15 [–.15, .42]	–.21 [–.43, .04]	.24 [–.04, .45]

PROMIS-D	–.04 [–.35, .25]	.26 [–.03, .53]	.16 [–.17, .48]	–.21 [–.60, .08]	.07 [–.25, .40]					

**MODEL 4** **ORL ESTIMATES (MODELED JOINTLY ACROSS SESSIONS)**										

***SELF-REPORT COLLECTED AT SAME SESSION***	**SESSION 1**					**SESSION 2**				
	
	***A+***	***A-***	***K***	***βf***	***βp***	***A+***	***A-***	***K***	***βf***	***βp***

BAS Total	.00 [–.33, .31]	–.13 [–.48, .19]	.04 [–.28, .34]	–.12 [–.41, .19]	.10 [–.24, .40]	–.02 [–.30, .24]	.09 [–.19, .38]	.15 [–.19, .46]	–.08 [–.36, .20]	.26 [–.03, .50]

BAS Drive	–.01 [–.36, .33]	–.22 [–.53, .12]	.01 [–.30, .31]	–.11 [–.41, .17]	.05 [–.28, .36]	.04 [–.24, .33]	.02 [–.27, .32]	.10 [–.19, .39]	–.02 [–.28, .25]	.22 [–.08, .48]

BAS Fun	.07 [–.22, .36]	.03 [–.31, .32]	.09 [–.19, .35]	–.06 [–.33, .21]	.12 [–.17, .38]	–.01 [–.25, .27]	.16 [–.13, .45]	.18 [–.16, .50]	.05 [–.28, .33]	.19 [–.11, .43]

BAS Reward Responsivity	–.05 [–.36, .24]	–.13 [–.43, .19]	.00 [–.33, .31]	–.13 [–.43, .15]	.09 [–.21, .37]	–.10 [–.37, .14]	.02 [–.24, .30]	.06 [–.24, .35]	–.20 [–.47, .08]	.19 [–.08, .40]

BIS Total	–.07 [–.35, .23]	–.18 [–.46, .12]	–.28 [–.55, .09]	–.24 [–.53, .03]	–.16 [–.41, .18]	.00 [–.34, .32]	.00 [–.24, .24]	–.04 [–.32, .27]	–.28 [–.54, .00]	.00 [–.25, .29]

PANAS PA	–.01 [–.27, .27]	–.15 [–.41, .13]	.07 [–.24, .38]	–.14 [–.40, .20]	.15 [–.14, .43]	**.27 [.02, .49]**	.02 [–.28, .34]	–.16 [–.42, .14]	–.13 [–.34, .13]	.01 [–.33, .36]

PANAS NA	.24 [–.04, .52]	.05 [–.22, .29]	–.08 [–.37, .27]	–.28 [–.52, .01]	.05 [–.23, .31]	**.41 [.15, .62]**	.11 [–.17, .36]	.01 [–.30, .33]	–.11 [–.49, .15]	.05 [–.20, .34]

MASQ General Distress Anxious	.20 [–.10, .46]	.20 [–.10, .44]	–.18 [–.43, .14]	–.12 [–.36, .18]	–.10 [–.36, .18]					

MASQ Anxious Arousal	.28 [–.01, .55]	.22 [–.06, .44]	–.17 [–.43, .20]	–.08 [–.34, .31]	–.15 [–.40, .14]					

MASQ General Distress Depressive	.30 [–.02, .56]	**.40 [.17, .63]**	–.06 [–.37, .27]	.01 [–.45, .35]	–.15 [–.44, .19]					

MASQ Anhedonic Depression	.00 [–.29, .29]	.21 [–.06, .50]	–.01 [–.31, .29]	.05 [–.31, .32]	–.10 [–.40, .21]					

SHAPS	–.13 [–.41, .25]	–.11 [–.40, .18]	.17 [–.16, .46]	–.13 [–.38, .15]	**.30 [.02, .52]**	–.14 [–.40, .13]	.06 [–.24, .35]	.11 [–.17, .45]	–.24 [–.45, .02]	.22 [–.06, .44]

PROMIS-D	.16 [–.18, .45]	**.31 [.05, .54]**	.05 [–.24, .39]	–.17 [–.60, .11]	.00 [–.28, .34]					


^a^ At session 1, the n for MASQ and PROMIS-D correlations is 46; the n for all other session 1 correlations is 48. ^b^ At session 2, the n for all correlations is 46.

The Model 3 ORL estimates showed a few moderate correlations with self-report measures. At session 1, Punishment Learning Rate (*A*-; more volatile updating for losses) was positively associated with the MASQ General Depressive subscale (*r* = .28, BCa 95% CI [.02, .60]) and Win Frequency Sensitivity (β*f*; sensitivity to win frequency irrespective of win magnitude) was negatively associated with state-level Negative Affect (*r* = –.29, BCa 95% CI [–.52, –.01]). Also at session 1, lower Memory Decay (*K*; less forgetting; remembering a longer sequence of deck selections) showed a moderate association with greater behavioral inhibition (*r* = –.32, BCa 95% CI [–.59, .08]); however, the confidence interval included zero. At session 2, Reward Learning Rate (*A*+; more volatile updating for rewards) was positively associated with both state-level Positive Affect (*r* = .31, BCa 95% CI [.03, .50]) and state-level Negative Affect (*r* = .40, BCa 95% CI [.16, .60]).

The Model 4 ORL estimates showed some additional associations with internalizing symptoms compared to Model 3 estimates. At session 1, Punishment Learning Rate was positively associated with both the MASQ General Depressive subscale (*r* = .40, BCa 95% CI [.17, .63]) and PROMIS Depression (*r* = .31, BCa 95% CI [.05, .54]). Session 1 Reward Learning Rate also showed some moderate positive correlations with several internalizing symptom measures and with state-level Negative Affect; however, all of the confidence intervals for these associations included zero. Also at session 1, Perseveration Tendency (β*p*; choice consistency irrespective of outcomes) showed a moderate positive association with SHAPS Anhedonia (reverse coded; *r* = .30, BCa 95% CI [.02, .52]), such that greater choice consistency was associated with lower anhedonia. Mirroring results for the Model 3 estimates, at session 2, Reward Learning Rate was positively associated with both state-level Positive Affect (*r* = .27, BCa 95% CI [.02, .49]) and state-level Negative Affect (*r* = .41, BCa 95% CI [.15, .62]). See Supplemental Tables 6 and 7 for construct validity correlations, excluding the participant with the outlier β*p* score. See Supplemental Table 9 for associations between Session 1 self-report and Session 2 IGT metrics.

## Discussion

Poor psychometric properties for behavioral tasks in general (Cooper et al., 2017; Hedge et al., 2018; Rouder & Haaf, 2019) and the IGT specifically (Buelow & Suhr, 2009; Schmitz, et al., 2020) limit their utility for individual difference research. We hypothesized, and found, that full generative modeling, compared to the traditional summary approach, yielded behavioral estimates with improved utility for individual differences research. More specifically, we demonstrated that a full generative model for the IGT increased test-retest reliability and also found preliminary evidence that this modeling strategy may enhance the task’s validity for characterizing internalizing pathology. Our full generative model incorporated both (1) the ORL computational model (Haines, et al., 2018) at the person-level and (2) at the group-level, parameters that explicitly modeled test-retest reliability for each of the ORL parameters, along with other group-level effects (e.g., group-level priors for each parameter). At the person-level, the ORL model decomposes observable IGT performance into distinct psychological processes (see Supplemental Discussion for parameter overview) that together give rise to observable choice behavior on the IGT. At the group-level, our full generative ORL model substantially improved test-retest reliability for each of the ORL parameters, in line with previous work demonstrating that generative models that incorporate hierarchical estimates of test-retest can strengthen test-retest correlations (Haines, et al., 2020). Further, unlike the IGT summary score, ORL parameters from the full generative model showed associations with internalizing symptom measures. Specifically, increased Punishment Learning Rate was associated with higher depression scores and Perseveration Tendency was associated with lower anhedonia scores.

### Full generative model improves test-retest reliability

Test-retest was only substantially improved in the full generative model. A skeptic may wonder if these improvements are due to HBA artificially inflating test-retest reliability. We believe this concern is not warranted for two reasons. First, the low reliability of Model 2 demonstrates that the use of a hierarchical model, in and of itself, does not necessarily improve test-retest reliability. Second, despite sharing the same priors, test-retest across the five ORL parameters in Model 4 showed considerable heterogeneity. Additionally, the use of the more theoretically informed person-level model, in and of itself, did not substantially improve test-retest for most ORL parameters (compared to summary score reliabilities). Together, these findings demonstrate enhanced test-retest reliability is not an artifact or deterministic feature of this joint HBA modeling strategy, but critically depends on *both* a joint hierarchical model and a well-specified person-level model, here the ORL. Further, our findings bolster previous work (Haines, et al., 2020), demonstrating that seemingly unreliable behavior may be highly reliable with a better specified model.

### ORL model provides insight into task functioning

In line with previous findings, we found the IGT summary score was associated with trait-motivation and state-mood (Buelow & Suhr, 2013; Case & Olino, 2020; Suhr & Tsanadis, 2007; van Honk, et al., 2002). Specifically, worse overall performance was associated with greater trait-level drive for desired goals and more intense mood, both positive and negative. While these associations are a bit difficult to interpret, application of the ORL may provide some insight into the common finding that negative mood is associated with lower IGT performance. Comparing findings from summary scores to those from the ORL estimates suggests that more intense moods (either positive or negative) may negatively influence performance through increased Reward Learning Rate, itself associated with worse overall performance.

Another finding to emerge was some inconsistent associations between performance and self-report across sessions. The summary score was negatively associated with trait drive only at session 1. Positive and negative mood were negatively associated with the summary score and positively associated with Reward Learning Rate only at session 2. These inconsistencies across sessions raise further questions about not only quantitative but also potential qualitative differences in learning/decision-making as individuals engage with an experience-based learning task multiple times. Application of the ORL model may help elucidate such potential changes at the process-level. For example, Reward and Punishment Learning Rates appeared to operate somewhat differently across testing sessions. In session 1, good performance was negatively associated with Reward Learning Rate and positively associated with Punishment Learning Rate. While associations remained directionally consistent across sessions, the association with good performance was strengthened for Reward Learning Rate but attenuated for Punishment Learning Rate at the subsequent session. This suggests there may be carry-over learning effects from the first testing session for some individuals, which is supported by the increased standard deviation in group-level task performance between sessions 1 and 2. Such carry-over learning may partially explain inconsistent associations between performance and self-report. Potential extensions/ adaptations to the current model may resolve such inconsistent associatons and/or address potential carry-over learning effects across sessions (see Supplemental section ‘Potential model adaptations to address outstanding issues’).

### Full generative modeling may enhance relevance of IGT for internalizing pathology

Previous work using IGT summary scores has shown mixed associations in relation to internalizing (Paulus & Yu, 2012), with depression and anxiety linked to both impaired (Alacreu-Crespo, et al., 2020; Cella, et al., 2010; Miu, Heilman, & Houser, 2008; Moniz, et al., 2016; Must, et al., 2006; Rinaldi, et al., 2020) and enhanced (Byrne et al., 2016; Mueller, et al., 2010; Smoski, et al., 2008) performance. Given that ORL parameters provide more refined indices of behavioral performance and given strong theoretical relevance of RL models for internalizing disorders (Bishop & Gagne, 2018; Chen, et al., 2015), we expected the ORL models would enhance construct validity in relation to internalizing symptoms. Indeed, the ORL models, particularly estimates from the full generative model, showed associations with internalizing symptom measures where the summary scores did not. Specifically, a higher Punishment Learning Rate was associated with greater depression scores on both the MASQ General Depressive and PROMIS Depression scales. As previous studies have generally found no difference in or lower learning rates associated with depression (Brown, et al., 2021; Chen, et al., 2015; Huys, et al., 2013), it is interesting that we found the opposite association in the first study we are aware of to examine IGT learning rates specifically in relation to internalizing. As optimal learning rate depends on task structure (Bishop & Gagne, 2018; Nussenbaum & Hartley, 2019), it may be interesting to examine learning rates (and other parameters) across a variety of tasks to understand relationships between learning rates across different environments and internalizing symptoms. We also found a higher Perseveration Tendency, or greater choice consistency irrespective of outcomes, was associated with lower anhedonia on the Snaith-Hamilton. Together with previous work demonstrating a link between reduced choice consistency and depression and suicidality (Alacreu-Crespo, et al., 2020; Dombrovski & Hallquist, 2021), our results suggest that reduced choice consistency may be specifically related to anhedonia symptoms, which are predictive of both depression (Khazanov & Ruscio, 2016) and suicidal ideation, controlling for depression (Ducasse, et al., 2018). Across both ORL models, Reward Learning Rate and Memory Decay parameters showed only non-significant associations with self-report measures, which may reflect a lack of power and/or simply a lack of association between fine-grained process-level metrics and coarser self-report measures. Given the lack of consensus across previous work examining summary IGT performance and internalizing, the current work demonstrates potential for full generative modeling to enhance the utility of the IGT for characterizing specific process-level learning/ decision-making patterns linked to internalizing symptoms. All behavior/self-report associations were inferred from a conservative modeling approach and should be interpreted as such.

### Limitations and future directions

This work has several notable limitations. First, we used a relatively small convenience sample and no psychopathology-related selection criteria were imposed. Thus, it will be important to replicate current results in a larger sample with higher symptom levels. Further, in translating this research clinically, it will be critical to include relevant clinical groups. Second, as there was some evidence that people may engage with the IGT differently across multiple sessions, future research should continue to develop generative models that can address potential quantitative and qualitative differences in learning/decision-making as individuals engage with the IGT multiple times. Future work should also examine whether administrations of the IGT beyond two time points show improved stability of behavioral estimates and stronger associations with self-report. Another limitation is that the current work cannot definitively demonstrate that associations between ORL model parameters and internalizing symptoms are due to improved psychometrics rather than other factors. Further, associations between ORL parameters and self-report were somewhat inconsistent across time. Future research could address this issue by incorporating self-report measures directly within the hierarchical models (e.g., Kildahl, et al., 2020; Kvam, et al., 2021; Vandekerckhove, 2014) to more precisely estimate associations between behavior and self-reported phenomenology. As we found differences in associations with state-, symptom-, and trait-level measures based on the modeling strategy used, future work could directly incorporate self-report constructs in models that simultaneously estimate behavioral metrics in either separate or joint estimations over time to explore factors that may represent underlying propensity for psychological pathology versus factors that may represent more temporally proximal markers of distress.

### Conclusions and broader implications

Overall, we demonstrate that full generative modeling of the IGT provides a set of performance metrics that are both richer in information and far more reliable across time than the traditional analytic approach. While we focused on the IGT and ORL model in particular, the current study has broader implications for experience-based learning and decision-making more generally. Many popular behavioral tasks demonstrate robust and reproducible results at the group-level; however, these tasks fall short when used for individual differences research, largely due to the low reliability of task metrics at the person-level (Cooper et al., 2017; Hedge et al., 2018). The current work demonstrates that even complex behavioral tasks can be used for individual differences research; however, extracting informative and reliable task metrics likely requires going beyond the simple summary statistics. This work joins a growing body of research demonstrating that hierarchical models that account for measurement uncertainty both between and within individuals can greatly improve test-retest reliability and optimize the utility of tasks for individual differences research (Brown, et al., 2021; Chen, et al., 2021; Haines, et al., 2020; Kildahl, et al., 2020; Kvam, et al., 2021; Rouder & Haaf, 2019; Vandekerckhove, 2014). As we did not find improvements in reliability or validity for the generative model utilizing a simple person-level model, this work further demonstrates the importance of a well-specified person-level model. Finally, our findings highlight the potential for well-specified generative models to support theory development and translation to applied work through the identification of lower-level learning and decision-making mechanisms that may underlie psychopathology.

## Additional File

The additional file for this article can be found as follows:

10.5334/cpsy.89.s1 Supplement. Supplemental Method and Results.
