# Supplementary material for: Enhancing the Psychometric Properties of the Iowa Gambling Task Using Full Generative Modeling
Source: Comput Psychiatr. 2022 Aug 26;6(1):189–212. doi: 10.5334/cpsy.89 (PMC10275579; doi:10.5334/cpsy.89)
Supplement: Supplement. — Supplemental Method and Results. [file cpsy-6-1-89-s1.pdf]

Supplement

Supplemental Background

Studies using computational modeling with the IGT

We are only aware of two studies that have used reinforcement learning models with the IGT to examine internalizing pathology. Byrne and colleagues (2016) found depression was positively associated with greater loss aversion in an undergraduate sample. Alacreu-Crespo and colleagues (2020) found that depressed patients had lower perseveration (choice consistency) than healthy controls, and that depressed patients with a previous suicide attempt had a higher learning rate (higher learning rate was also associated with worse overall performance) and had lower loss aversion compared to healthy controls.

Supplemental Method

Supplemental Table 1. Win/Loss Contingencies for Decks in the IGT.

Deck	**A**	**B**	**C**	**D**
Gain (presented every trial)	$100	$100	$50	$50
Losses (occurring every 10 trials)	-$150	-$1,250	-$25	-$250
	-$200		-$50	
	-$250		-$75	
	-$300			
	-$350			
Net value across 10 trials	-$250	-$250	$350	$250

Supplemental Table 2. In-Sample Reliabilities.

In-Sample Reliabilities for Self-Report Measures

	Session 1 α	Session 2 α
Behavioral Inhibition (BIS)	.81	.78
Behavioral Activation Total (BAS-Total)	.88	.76
BAS-Drive	.80	.64
BAS-Fun Seeking	.76	.57
BAS-Reward Responsiveness	.84	.68
PANAS Positive Affect	.90	.80
PANAS Negative Affect	.91	.88
MASQ-General Distress Anxiety	.86	NA
MASQ-General Distress Depression	.95	NA
MASQ-Anxious Arousal	.89	NA
MASQ-Anhedonic Depression	.80	NA
Snaith-Hamilton Pleasure Scale	.88	.88
PROMIS-Depression	.98	NA

Supplemental Table 3. Descriptive statistics for self-report measures.

	Session 1 (n = 46 or 48)			Session 1 (n = 46)		
	mean	*(sd)*	range	mean	*(sd)*	range
BAS Total	43	*(6.0)*	30 – 52	42	*(4.6)*	31 – 52
BAS Drive	12	*(2.5)*	6 – 16	12	*(2.1)*	8 – 16
BAS Fun	13	*(2.3)*	9 – 16	12	*(1.8)*	10 – 16
BAS Reward Responsivity	18	*(2.3)*	12 – 20	17	*(2.2)*	10 – 20
BIS Total	21	*(3.9)*	10 – 28	21	*(3.6)*	11 – 28
PANAS PA	24	*(8.7)*	12 – 45	24	*(6.7)*	14 – 45
PANAS NA	16	*(6.5)*	10 – 36	17	*(6.4)*	10 – 38
MASQ General Distress Anxious	21	*(8.2)*	11 – 38			
MASQ Anxious Arousal	27	*(10.0)*	17 – 55			
MASQ General Distress Depressive	24	*(11.0)*	12 – 47			
MASQ Anhedonic Depression	34	*(14.0)*	9 – 62			
SHAPS	5.2	*(4.1)*	0 – 14	4.7	*(4.1)*	0 – 14
PROMIS-D	32	*(24.7)*	4 – 93			

Model 4 Specification

Model 4 assumed that person-level parameters across sessions followed from group-level multivariate normal distributions, where each separate parameter had its own multivariate normal distribution in which it was assumed that the priors for locations (i.e., means) and scales (i.e., standard deviations) were assigned normal distributions. Here, for conceptual clarity, we present the centered parameterization of Model 4 parameters (and below in the subsection labeled 'Group-level Re-parameterizations to Increase Efficiency' we present the mathematically-equivalent, non-centered parameterization, which was used to make MCMC sampling more efficient). Using Punishment Learning Rate as an example, formal specification of the bounded parameters followed the form:

$$\mu_{\mathrm{A}_{-},t} \sim \text{Normal}(0,1)$$

$$\sigma_{\mathrm{A}_{-},t} \sim \text{Normal}(0,.2)$$

$$\begin{bmatrix} \mathrm{A}'_{-,1} \\ \mathrm{A}'_{-,2} \end{bmatrix} \sim \text{MVNormal}\left(\begin{bmatrix} \mu_{\mathrm{A}_{-},1} \\ \mu_{\mathrm{A}_{-},2} \end{bmatrix}, \mathbf{S}_{A_{-}}\right)$$

$$\mathrm{A}_{-,t} = \Phi\left(\mathrm{A}'_{-,t}\right)$$

where $\mu_{\mathrm{A}_{-},t}$ and $\sigma_{\mathrm{A}_{-},t}$ are the location and scale parameters for the group-level distributions for each session t, $\mathrm{A}'_{-,t}$ is a vector of individual-level parameters for session t on the unconstrained space, and $\mathrm{A}_{-,t}$ is a vector of the same individual-level parameters after they have been transformed to the appropriate bounds. This parameterization both ensures that the hyper prior distribution over the subject-level parameters is (near)uniform between the parameter bounds and allows for us to use multivariate normal distributions to model the correlation between bounded parameters. For example, $A_{-_{i,1}}$ and $A_{-_{i,2}}$ are the Punishment Learning Rates for person i at both session 1 and 2, respectively. $\Phi(\dots)$ is the cumulative distribution function for the standard normal distribution, which is used because the person-level learning rates must fall between 0 and 1. Because $\Phi(\dots)$ transforms from $[-\infty, +\infty] \rightarrow [0,1]$, it allows for us to use the multivariate normal group-level distribution to capture the test-retest correlation despite the multivariate normal distribution itself having support outside of [0,1].

For parameters bounded $\in (0, \text{upper})$ (e.g., K), we used the same parameterization as above but scaled to the upper bound accordingly:

$$K_t \sim \Phi(K'_t) \cdot 5$$

For unbounded parameters (β_f and β_p), we used the same parameterization outlined above except we set the hyper standard deviations (e.g., $\sigma_{\beta_p,t}$) to a half-Cauchy(0, 1). Because these parameters are unbounded, they follow directly from a multivariate normal distribution and do not require transformations.

For brevity, we can alternatively write out the probabilistic model with inverse parameter transformations on the left-hand side of the equation. Doing so, the multivariate normal distribution for each parameter was as follows:

Reward Learning Rate $A_{+,t}$

$$\begin{bmatrix} \Phi^{-1}(A_{+_{i,1}}) \\ \Phi^{-1}(A_{+_{i,2}}) \end{bmatrix} \sim \text{MVNormal}\left(\begin{bmatrix} \mu_{A_+,1} \\ \mu_{A_+,2} \end{bmatrix} , \mathbf{S}_{A_+} \right)$$

Punishment Learning Rate $A_{-,t}$

$$\begin{bmatrix} \Phi^{-1}(A_{-_{i,1}}) \\ \Phi^{-1}(A_{-_{i,2}}) \end{bmatrix} \sim \text{MVNormal}\left(\begin{bmatrix} \mu_{A_-,1} \\ \mu_{A_-,2} \end{bmatrix} , \mathbf{S}_{A_-} \right)$$

Memory Decay K_t

$$\begin{bmatrix} \Phi^{-1}(K_{i,1}/5) \\ \Phi^{-1}(K_{i,2}/5) \end{bmatrix} \sim \text{MVNormal}\left(\begin{bmatrix} \mu_{K,1} \\ \mu_{K,2} \end{bmatrix} , \mathbf{S}_{K} \right)$$

Win Frequency Sensitivity $\beta_{f,t}$

$$\begin{bmatrix} \beta_{f\ i,1} \\ \beta_{f\ i,2} \end{bmatrix} \sim \text{MVNormal}\left(\begin{bmatrix} \mu_{\beta_f,1} \\ \mu_{\beta_f,2} \end{bmatrix} , \mathbf{S}_{\beta_f} \right)$$

Perseveration Tendency $\beta_{p,t}$

$$\begin{bmatrix} \beta_{p_{i,1}} \\ \beta_{p_{i,2}} \end{bmatrix} \sim \text{MVNormal}\left(\begin{bmatrix} \mu_{\beta_p,1} \\ \mu_{\beta_p,2} \end{bmatrix} , \mathbf{S}_{\beta_p} \right)$$

Above, $\Phi^{-1}(\dots)$ is the inverse of the cumulative probability distribution for the standard normal distribution (i.e. the quantile function), which transforms from $[0,1] \rightarrow [-\infty, +\infty]$.

Each parameter's multivariate normal distribution contains a covariance matrix. Again, using Punishment Learning Rate as an example, $\mathbf{S}_{A\_}$ is a covariance matrix that captures the correlation between the person-level parameters across sessions. Specifically, $\mathbf{S}_{A\_}$ can be decomposed into the group-level standard deviations at each session ($\sigma_{A\_,1}$ and $\sigma_{A\_,2}$) and the 2x2 correlation matrix of interest, $\mathbf{R}_{A\_}$:

$$\mathbf{S}_{A\_} = \begin{pmatrix} \sigma_{\mathrm{A\_},1} & 0 \\ 0 & \sigma_{\mathrm{A\_},2} \end{pmatrix} \mathbf{R}_{A\_} \begin{pmatrix} \sigma_{\mathrm{A\_},1} & 0 \\ 0 & \sigma_{\mathrm{A\_},2} \end{pmatrix}$$

Here, $\mathbf{R}_{A\_}$ is a correlation matrix with one free parameter (ρ) on the off-diagonal:

$$\mathbf{R}_{A\_} = \begin{pmatrix} 1 & \rho_{A\_} \\ \rho_{A\_} & 1 \end{pmatrix}$$

where the free parameter $\rho_{A\_}$ indicates the test-retest correlation of interest—the value that we present throughout the text. Finally, we assume the following LKJ prior on each correlation matrix:

$$\mathbf{R}_{A\_} \sim \mathrm{LKJcorr}(1)$$

With only a single free parameter, this prior equates to a uniform distribution between -1 and 1 on ρ, meaning that all possible values for the test-retest correlation were assumed to be equally likely.

Group-level Re-parameterizations to Increase Efficiency

Here, we present re-parameterizations employed to make the MCMC sampling more efficient (see Stan code available on OSF: https://osf.io/b3kwz/?view_only=fea448b0bcff48a2bc0304e7b4448720). These re-parameterizations are mathematically-equivalent to the centered parameterizations presented above and included non-centered parameterizations for the hierarchical (group-level) components of the model, along with Cholesky decompositions to re-parameterize the test-retest correlation matrices, which makes sampling from the multivariate normal distribution more efficient. For individual-level parameters drawn from multivariate normal distributions, which we used to estimate test-retest correlations, we used a Cholesky decomposition to employ non-centered parameterizations. Specifically, a covariance matrix $\mathbf{S}$ can be decomposed into its Cholesky factor $L_{\mathbf{S}}$, where $\mathbf{S}$ = $L_{\mathbf{S}}L_{\mathbf{S}}^{T}$. Then, individual-level parameters drawn from independent, standard normal distributions can be correlated using the Cholesky factor $L_{\mathbf{S}}$, as shown below. The Cholesky factor ($L_{\mathbf{S}}$) of the covariance matrix $\mathbf{S}$ is equal to the diagonal matrix of the group-level *SD*s multiplied by the Cholesky factor of the correlation matrix $L_{\mathbf{R}}$. Therefore, as opposed to sampling directly from a multivariate normal distribution to estimate a correlation matrix $\mathbf{R}$, we can sample from the group-level means, standard deviations, the Cholesky factor of the correlation matrix ($L_{\mathbf{R}}$), and individual-level parameters (e.g., $A'_{-i,1}$ and $A'_{-i,2}$) independently and then reconstruct the correlation matrix afterward as $\mathbf{R}$ = $L_{\mathbf{R}}L_{\mathbf{R}}^{T}$.

Using Punishment Learning Rate as an example, the non-centered parameterization was as follows:

$$\mu_{\mathrm{A\_},t} \sim \mathrm{Normal}(0,1)$$

$$\sigma_{\mathrm{A\_},t} \sim \mathrm{Normal}(0,.2)$$

$$\mathrm{A'}_{-,i,t} \sim \mathrm{Normal}(0,1)$$

$$L_{\mathbf{R}_\mu} \sim \mathrm{LKJcorr}(1)$$

$$L_{\mathbf{S}_\mu} = \begin{pmatrix} \sigma_{\mathrm{A}_-,1} & 0 \\ 0 & \sigma_{\mathrm{A}_-,2} \end{pmatrix} L_{\mathbf{R}_\mu}$$

$$\begin{bmatrix} A_{-i,1} \\ A_{-i,2} \end{bmatrix} = \Phi(\begin{bmatrix} \mu_{A_-,1} \\ \mu_{A_-,1} \end{bmatrix} + \ L_{\mathbf{S}_\mu} \begin{bmatrix} A'_{-i,1} \\ A'_{-i,2} \end{bmatrix})$$

$$\mathbf{R}_\mu = L_{\mathbf{R}_\mu} L_{\mathbf{R}_\mu}^T$$

Model validation

Parameter Recovery. The ORL model was developed by Haines and colleagues (2018) to be used for making inference on individual differences related to clinical phenomena. As part of model development, parameter recovery was conducted for the ORL model and competing computational models, and the ORL was found to have good recovery of both parameter means and of the full posteriors compared to competing models (Haines, et. al., 2018). We also performed parameter recovery in the current sample. Data was simulated using parameters set to known values and the ORL model was subsequently fit to the simulated data to "recover" the parameter values. Correlations between known and recovered parameters, or "recovery statistics" represent model performance and the reliability of parameters, with values closer to r =1 indicating more precise person-level measures. When the ORL model was fit to data from the original IGT (single administration) for 200 simulated subjects, recovery statistics were acceptable across all parameters (*A+ r* =.81; *A- r* =.77; *K r* =.52; β*f r* = .84; β*p r* = .95).

Posterior Predictive Checks. To ensure that the models fit the data well, we performed posterior predictive checks in which a given model is used to simulate data, and the simulated data is subsequently compared to the observed data. More specifically, fitted model parameters were used to simulate data and the simulated data was plotted against the observed data as a qualitative check for model fit.

Supplemental Figure 1. Posterior Predictive Check for Model 2. Simulated data using Model 2 estimates of θ reproduced the Model 1 observed summary score at the person-level for both Session 1 and Session 2. Note that four participants from Session 1 were not present for Session 2, which we indicate in the Session 2 panel with points at 0%. Despite not having Session 2 data on these participants, the model predicts variation between these missing participants based on their Session 1 data.

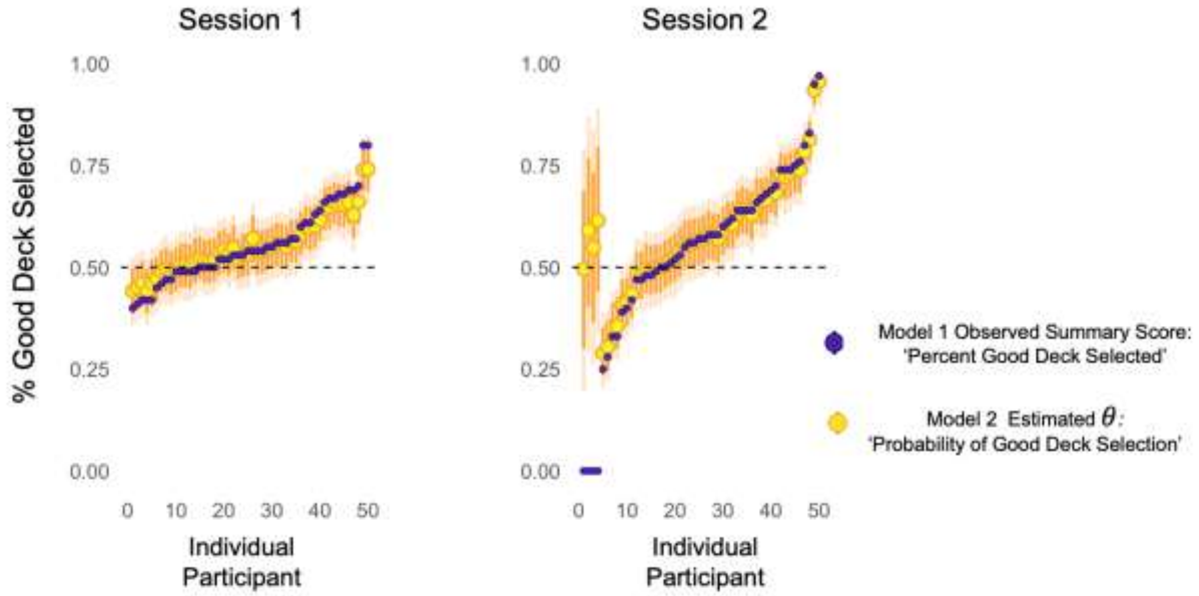

Supplemental Figure 2. Posterior Predictive Check for Model 3. Simulated data using Model 3 estimates of the five free ORL parameters reproduced observed group-averaged, trial-level choice behavior for both Session 1 and Session 2. Note: a random selection strategy would roughly produce a 25% group-averaged selection rate (marked by the horizontal red line) for each deck, on any given trial. 50% and 95% highest density intervals are demarcated by dark and light orange areas, respectively.

Session 1

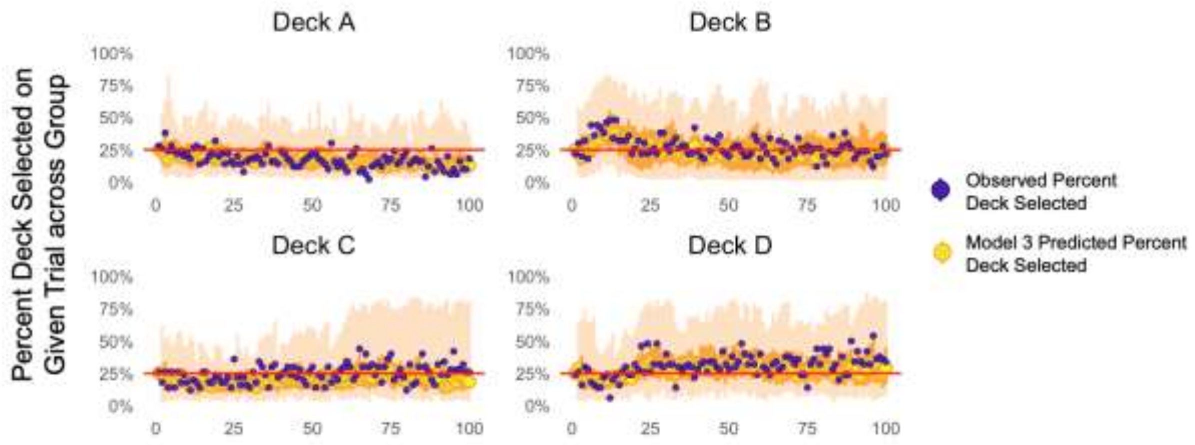

Session 2

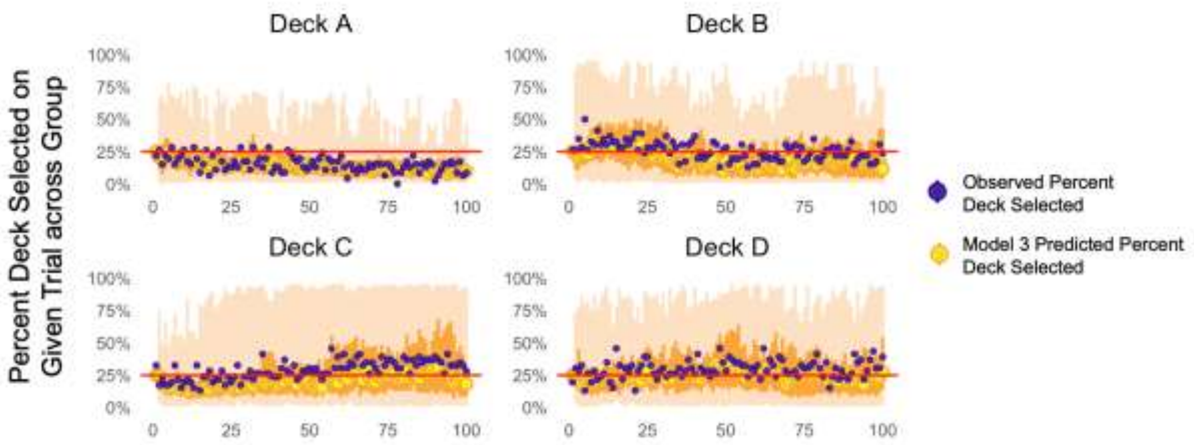

Supplemental Results

Performance across testing sessions

Using both 'summary score models', performance means were similar across sessions, while the standard deviation in performance was greater at session 2. Supplemental Figure 3A shows performance across the two sessions for the observed 'percent good deck selected' (Model 1). For Model 1, there was no significant difference in mean performance between sessions (session 1 M = 55.78; session 2 M = 58.63; Welch's $t(73) = -1.19$, 95% CI [-8.49, 2.15]), but the standard deviation in performance was significantly greater at session 2 (session 1 SD = 9.54; session 2 SD = 15.64; Levene's $F(1,94) = 7.74$, $p = .007$). For the Model 2 metric, θ (estimated probability of a good deck selection), the HDI plots in Supplemental Figure 3B show the difference in performance between the session 1 and session 2 posterior distributions for the mean (μ) and standard deviation (σ) of θ, respectively. For session 2 μ – session 1 μ, the 95% HDI indicates there is small-to-no difference in mean performance across sessions, with a posterior mean of .03 (95% CI = [-.02, .08]) for the distribution representing the difference in mean performance between sessions. However, the Model 2 difference in the standard deviation of performance across sessions had a posterior mean of .07 (95% CI = [.03, .11]) with a 95% HDI that does not include 0, indicating a larger standard deviation in session 2 performance. See Supplemental Table 4 for Model 3 and 4 parameter estimate posterior means. As shown in Supplemental Figure 4, there was no strong evidence of group-level between-session differences for the Model 3 parameters.

Supplemental Figure 3: Summary Score Performance Metrics across Sessions. (A) Model 1: Scatterplots showing observed 'percentage good deck selected' across sessions. (B) Model 2: Highest Density Interval (HDI) plots showing *differences* for the mean (μ) and standard deviation (σ) of θ, the estimated 'probability of a good deck selection'. Horizontal red lines indicate the 95% highest density intervals for estimates within the difference distributions (session 2 distribution – session 1 distribution) and vertical red lines indicate the posterior means for these difference estimations.

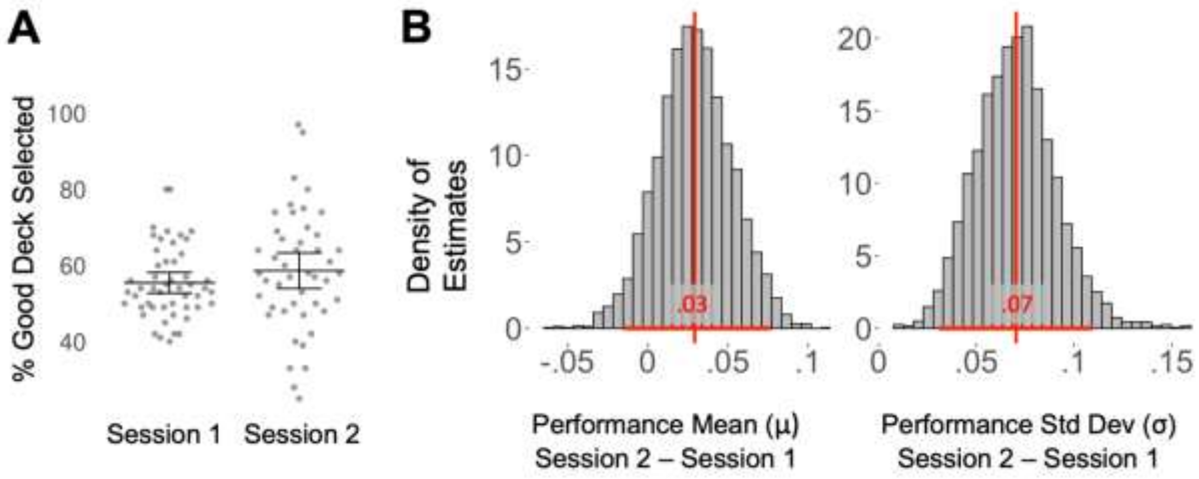

Supplemental Table 4. Posterior Means for Model 3 and Model 4 ORL Parameters

Model 3

	**Session 1** (n = 50)	**Session 2** (n = 46)
	Posterior Means	
A+	.38	.33
A-	.13	.12
K	.54	.81
β*f*	1.57	2.06
β*p*	-1.63	-.74

Model 4

	**Session 1** (n = 50)	**Session 2** (n = 50)
	Posterior Means	
A+	.34	.31
A-	.13	.12
K	.68	.85
β*f*	1.55	2.09
β*p*	-1.34	-.35

Supplemental Figure 4. Group-Level Performance Differences for the ORL Parameters between Testing Sessions. As all 95% CIs included zero, there was no strong evidence of group-level between-session differences for the Model 3 parameters.

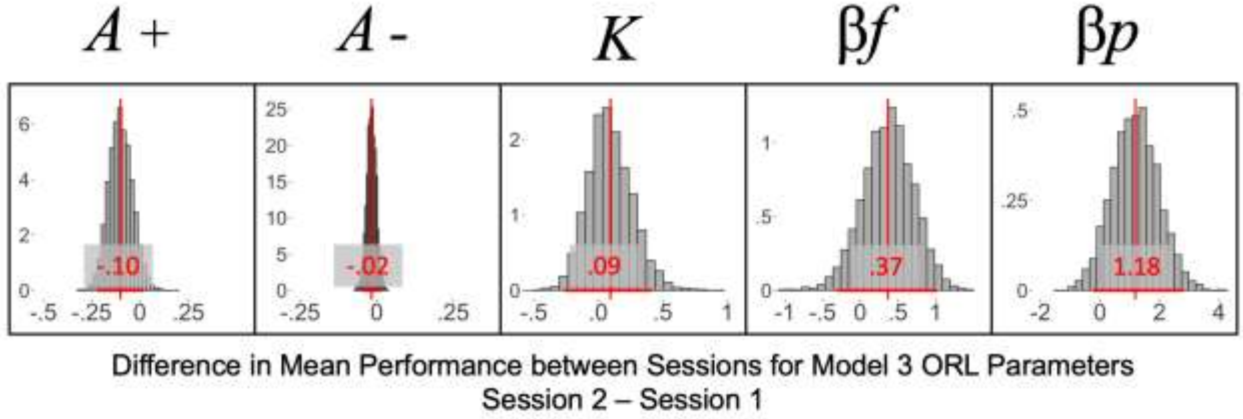

Supplemental Table 5. Associations between ORL Parameters and the Model 1 Summary Score Excluding Outlier. In the manuscript, the scatterplots in Figure 4B revealed an outlier on the β*p* parameter at Session 2. All correlations between the Model 3 and Model 4 ORL parameters and the Model 1 Summary Score are reported here with the outlier removed. Removal of the outlier did not substantially change correlation results for any parameter. CIs represent 95% bias-corrected and accelerated bootstrapped confidence intervals (95% BCa CIs).

	Model 3	
	**Session 1** (n = 49)	**Session 2** (n = 45)
	r 95% CI	*r* 95% CI
A+	-.49 [-.66,-.16]	-.67 [-.79,-.50]
A-	.57 [.30,.77]	.20 [-.07,.43]
K	.25 [-.01,.54]	.11 [-.18,.37]
β*f*	.26 [-.06,.54]	.28 [-.05,.52]
β*p*	.17 [-.29,.47]	.21 [-.12,.48]

	Model 4	
	**Session 1** (n = 49)	**Session 2** (n = 49)
	r 95% CI	*r* 95% CI
A+	-.40 [-.59,-.01]	-.63 [-.77,-.44]
A-	.49 [.21,.70]	.16 [-.13,.40]
K	.19 [-.09,.44]	.09 [-.21,.36]
β*f*	.31 [.02,.56]	.31 [.00,.55]
β*p*	.15 [-.25,.43]	.19 [-.10,.45]

Supplemental Table 6. Model 1 and Model 2 Correlations with Self-Report Measures: Excluding the Single Outlier Subject on the β*p* parameter at Session 2.

	Model 1		Model 2	
	Percentage Good Deck Selected		Probability of Good Deck Selection (θ)	
	Session 1	Session 2	Session 1	Session 2
Self-Report Collected at Same Session	(n = 45 or 47)	(n = 45)	(n = 45 or 47)	(n = 45)
BAS Total	-.25 [-.55, .03]	.07 [-.18, .37]	-.25 [-.54, .05]	.06 [-.19, .36]
BAS Drive	**-.39 [-.67, -.06]**	-.06 [-.36, .26]	**-.37 [-.67, -.04]**	-.07 [-.37, .25]
BAS Fun	-.10 [-.40, .20]	.12 [-.18, .39]	-.08 [-.39, .20]	.12 [-.17, .39]
BAS Reward Responsivity	-.15 [-.43, .13]	.12 [-.18, .39]	-.15 [-.44, .12]	.11 [-.19, .38]
BIS Total	-.20 [-.49, .09]	-.04 [-.34, .25]	-.18 [-.48, .12]	-.04 [-.35, .24]
PANAS PA	.02 [-.24, .26]	**-.32 [-.52, -.07]**	.00 [-.26, .24]	**-.32 [-.51, -.08]**
PANAS NA	-.13 [-.34, .07]	**-.41 [-.63, -.14]**	-.15 [-.34, .06]	**-.42 [-.63, -.16]**
MASQ General Distress Anxious	-.13 [-.39, .21]		-.14 [-.40, .21]	
MASQ Anxious Arousal	-.19 [-.45, .08]		-.21 [-.47, .07]	
MASQ General Distress Depressive	.01 [-.27, .37]		-.02 [-.30, .36]	
MASQ Anhedonic Depression	.11 [-.23, .49]		.11 [-.25, .48]	
SHAPS	.00 [-.25, .28]	.03 [-.21, .30]	.00 [-.24, .27]	.03 [-.21, .31]
PROMIS-D	.16 [-.17, .53]		.13 [-.20, .50]	

Supplemental Table 7. Model 3 and Model 4 Correlations with Self-Report Measures: Excluding the Single Outlier Subject on the βp parameter at Session 2.

Model 3										
	ORL Estimates (Modeled Separately at Each Session)									
	Session 1 (n = 45 or 47)					Session 2 (n = 45)				
Self-Report Collected at Same Session	***A+***	***A-***	***K***	**β*f***	**β*p***	***A+***	***A-***	***K***	**β*f***	**β*p***
BAS Total	.00 [-.32, .29]	-.19 [-.56, .14]	.02 [-.34, .35]	-.11 [-.42, .19]	.04 [-.29, .39]	.01 [-.26, .29]	.14 [-.12, .40]	.09 [-.23, .39]	-.07 [-.34, .20]	.16 [-.14, .39]
BAS Drive	.00 [-.38, .35]	**-.31 [-.63, .05]**	-.06 [-.39, .25]	-.07 [-.38, .21]	-.03 [-.38, .36]	.09 [-.20, .39]	.08 [-.17, .36]	.06 [-.22, .33]	-.03 [-.28, .20]	.12 [-.20, .38]
BAS Fun	.03 [-.26, .31]	-.03 [-.37, .27]	.09 [-.25, .37]	-.11 [-.37, .17]	.12 [-.21, .41]	.02 [-.23, .29]	.17 [-.11, .43]	.11 [-.22, .45]	.05 [-.27, .33]	.13 [-.19, .42]
BAS Reward Responsivity	-.02 [-.30, .25]	-.13 [-.45, .16]	.02 [-.35, .36]	-.10 [-.41, .18]	.02 [-.29, .32]	-.09 [-.35, .18]	.07 [-.19, .32]	.03 [-.27, .30]	-.16 [-.44, .10]	.11 [-.14, .35]
BIS Total	.03 [-.25, .30]	-.16 [-.47, .15]	**-.31 [-.59, .08]**	-.21 [-.51, .10]	-.18 [-.47, .19]	.00 [-.32, .31]	.08 [-.17, .30]	.02 [-.28, .34]	-.22 [-.50, .06]	-.02 [-.33, .30]
PANAS PA	-.10 [-.39, .16]	-.23 [-.46, .05]	.07 [-.27, .39]	-.12 [-.37, .23]	.11 [-.19, .39]	**.32 [.03, .51]**	.04 [-.26, .45]	-.23 [-.47, .06]	-.10 [-.32, .15]	-.14 [-.46, .17]
PANAS NA	.08 [-.19, .31]	-.10 [-.31, .12]	-.04 [-.36, .34]	-.29 [-.52, -.01]	.06 [-.23, .36]	**.40 [.15, .60]**	.13 [-.17, .39]	-.08 [-.33, .26]	-.04 [-.39, .24]	-.09 [-.31, .24]
MASQ General Distress Anxious	.07 [-.23, .35]	.10 [-.20, .37]	-.07 [-.35, .25]	-.16 [-.39, .15]	-.07 [-.35, .26]					
MASQ Anxious Arousal	.14 [-.13, .39]	.13 [-.13, .36]	-.06 [-.37, .31]	-.11 [-.36, .27]	-.11 [-.41, .20]					
MASQ General Distress Depressive	.03 [-.27, .29]	.26 [-.00, .57]	.07 [-.27, .44]	-.06 [-.48, .27]	-.16 [-.48, .16]					
MASQ Anhedonic Depression	-.06 [-.38, .27]	.20 [-.12, .53]	.02 [-.29, .39]	.01 [-.33, .28]	-.01 [-.34, .31]					
SHAPS	-.11 [-.39, .17]	-.14 [-.38, .13]	.11 [-.20, .42]	-.11 [-.35, .17]	.24 [-.05, .49]	-.15 [-.41, .13]	-.06 [-.32, .19]	.12 [-.19, .40]	-.25 [-.46, -.00]	.18 [-.13, .42]
PROMIS-D	-.03 [-.34, .26]	.27 [-.01, .54]	.16 [-.16, .48]	-.21 [-.59, .08]	.08 [-.25, .42]					

Model 4										
	ORL Estimates (Modeled Jointly across Sessions)									
	Session 1 (n = 45 or 47)					Session 2 (n = 45)				
Self-Report Collected at Same Session	***A+***	***A-***	***K***	**β*f***	**β*p***	***A+***	***A-***	***K***	**β*f***	**β*p***
BAS Total	-.02 [-.36, .29]	-.15 [-.51, .19]	.05 [-.28, .35]	-.12 [-.43, .18]	.03 [-.28, .36]	-.04 [-.32, .22]	.06 [-.22, .36]	.12 [-.22, .45]	-.10 [-.39, .17]	.18 [-.13, .42]
BAS Drive	-.03 [-.41, .32]	-.24 [-.56, .10]	.02 [-.30, .32]	-.11 [-.42, .17]	-.01 [-.33, .33]	.02 [-.27, .33]	-.01 [-.30, .30]	.07 [-.22, .36]	-.04 [-.31, .23]	.13 [-.18, .40]
BAS Fun	.06 [-.24, .33]	.02 [-.33, .31]	.10 [-.19, .36]	-.06 [-.33, .21]	.08 [-.23, .37]	-.02 [-.27, .26]	.15 [-.15, .43]	.16 [-.18, .49]	.04 [-.29, .33]	.15 [-.17, .43]
BAS Reward Responsivity	-.07 [-.39, .21]	-.15 [-.45, .16]	.00 [-.33, .31]	-.14 [-.44, .15]	.02 [-.28, .32]	-.12 [-.39, .13]	.00 [-.25, .28]	.04 [-.26, .35]	-.22 [-.49, .06]	.12 [-.13, .37]
BIS Total	-.07 [-.35, .23]	-.18 [-.46, .12]	-.28 [-.55, .10]	-.24 [-.54, .03]	-.15 [-.43, .20]	.00 [-.34, .31]	.00 [-.24, .24]	-.05 [-.33, .26]	-.28 [-.55, .00]	.00 [-.31, .34]
PANAS PA	-.03 [-.30, .26]	-.18 [-.43, .11]	.08 [-.23, .40]	-.15 [-.40, .21]	.10 [-.21, .38]	.26 [-.00, .48]	-.01 [-.31, .32]	-.19 [-.45, .10]	-.15 [-.37, .12]	-.12 [-.45, .18]
PANAS NA	.23 [-.06, .52]	.04 [-.23, .29]	-.07 [-.37, .29]	-.28 [-.52, -.01]	.02 [-.28, .31]	**.40 [.15, .62]**	.10 [-.19, .35]	-.01 [-.33, .32]	-.13 [-.49, .15]	-.02 [-.26, .32]
MASQ General Distress Anxious	.20 [-.10, .48]	.20 [-.09, .44]	-.18 [-.43, .14]	-.12 [-.36, .18]	-.11 [-.39, .17]					
MASQ Anxious Arousal	.28 [-.01, .58]	.23 [-.05, .45]	-.17 [-.44, .18]	-.08 [-.35, .30]	-.15 [-.44, .15]					
MASQ General Distress Depressive	.29 [-.05, .55]	**.38 [.15, .63]**	-.05 [-.36, .29]	.00 [-.46, .33]	-.23 [-.50, .12]					
MASQ Anhedonic Depression	.03 [-.28, .31]	.24 [-.03, .53]	-.02 [-.32, .30]	.06 [-.32, .33]	-.04 [-.35, .28]					
SHAPS	-.15 [-.44, .23]	-.14 [-.41, .16]	.18 [-.14, .47]	-.13 [-.38, .15]	.26 [-.04, .50]	-.16 [-.42, .12]	.03 [-.26, .33]	.09 [-.20, .42]	-.27 [-.48, -.00]	.16 [-.16, .41]
PROMIS-D	.17 [-.18, .45]	**.32 [.06, .55]**	.05 [-.24, .39]	-.17 [-.60, .12]	.03 [-.28, .36]					

Supplemental Table 8. Post-hoc correlations between Session 1 Self-Report Data and Session 1 and Session 2 IGT Metrics for Models 1 and 2. Session 1 correlations (session 1 IGT metrics and session 1 self-report) are the same as those reported in Table 2 of the main text and are replicated here for comparison with session 2 correlations (session 2 IGT metrics and session 1 self-report). Interestingly, there was a moderate negative association between the session 1 MASQ General Depressive subscale and the session 2 summary score for Model 2 (r = -.24, BCa 95% CI [-.44, -.01]).

	Model 1 Percentage Good Deck Selected		Model 2 Probability of Good Deck Selection (θ)	
Self-Report Collected at Session 1	Session 1	Session 2	Session 1	Session 2
BAS Total	-.25 [-.53, .05]	.03 [-.24, .34]	-.24 [-.52, .05]	-.01 [-.25, .28]
BAS Drive	**-.38 [-.66, -.05]**	.01 [-.28, .36]	**-.37 [-.66, -.03]**	-.04 [-.31, .31]
BAS Fun	-.10 [-.40, .20]	.10 [-.20, .39]	-.08 [-.38, .20]	.08 [-.21, .35]
BAS Reward Responsivity	-.14 [-.43, .13]	-.05 [-.30, .22]	-.14 [-.42, .12]	-.06 [-.30, .19]
BIS Total	-.20 [-.49, .09]	.00 [-.30, .37]	-.19 [-.48, .12]	.00 [-.29, .34]
PANAS PA	.02 [-.22, .25]	-.12 [-.38, .13]	.01 [-.25, .24]	-.09 [-.34, .14]
PANAS NA	-.13 [-.33, .07]	-.15 [-.33, .07]	-.14 [-.34, .05]	-.13 [-.31, .07]
MASQ General Distress Anxious	-.13 [-.39, .21]	-.15 [-.36, .13]	-.14 [-.40, .21]	-.17 [-.37, .12]
MASQ Anxious Arousal	-.19 [-.45, .08]	-.20 [-.41, .09]	-.21 [-.46, .07]	-.22 [-.41, .05]
MASQ General Distress Depressive	.01 [-.27, .39]	-.24 [-.42, -.00]	-.02 [-.29, .36]	**-.24 [-.44, -.01]**
MASQ Anhedonic Depression	.11 [-.23, .46]	-.01 [-.31, .27]	.11 [-.25, .47]	-.01 [-.30, .28]
SHAPS	.01 [-.24, .27]	.03 [-.25, .31]	.01 [-.24, .26]	.04 [-.23, .29]
PROMIS-D	.15 [-.16, .52]	-.20 [-.44, .05]	.12 [-.20, .49]	-.19 [-.43, .08]

a At session 1, the n for MASQ and PROMIS-D correlations is 46; the n for all other session 1 correlations is 48

b At session 2, for all Model 1 correlations n is 44; for Model 2, the n for MASQ and PROMIS-D correlations is 46 and the n for all other correlations is 48

Supplemental Table 9. Post-hoc correlations between Session 1 Self-Report Data and Session 1 and Session 2 IGT Metrics for Models 3 and 4. Session 1 correlations (session 1 IGT metrics and session 1 self-report) are the same as those reported in Table 3 of the main text and are replicated here for comparison with session 2 correlations (session 2 IGT metrics and session 1 self-report). Focusing on Model 4, results were fairly consistent with the associations between same-day ORL parameters and self-report, with moderate positive associations between session 2 Punishment Learning Rate and MASQ General Depressive subscale (r = .36, BCa 95% CI [.09, .56]) and session 2 Perseveration Tendency and SHAPS Anhedonia (r = .31, BCa 95% CI [.02, .50]). Unlike the session 1 results, session 2 Reward Learning Rate showed positive associations with state-level Negative Affect (r = .29, BCa 95% CI [.01, .60]), with MASQ Anxious Arousal (r = .31, BCa 95% CI [.01, .60]), and with the MASQ General Depressive subscale (r = .39, BCa 95% CI [.10, .60]).

Model 3

ORL Estimates (Modeled Separately at Each Session)

	Session 1					Session 2				
Self-Report Collected at Session 1	***A+***	***A-***	***K***	**β*f***	**β*p***	***A+***	***A-***	***K***	**β*f***	**β*p***
BAS Total	.03 [-.29, .30]	-.17 [-.52, .17]	.02 [-.35, .33]	-.11 [-.42, .19]	.08 [-.25, .41]	.03 [-.29, .36]	.01 [-.32, .32]	.09 [-.21, .36]	-.14 [-.43, .17]	.23 [-.05, .44]
BAS Drive	.02 [-.34, .36]	-.29 [-.60, .07]	-.05 [-.39, .24]	-.07 [-.38, .20]	.01 [-.33, .38]	.04 [-.29, .35]	.00 [-.28, .30]	.05 [-.24, .35]	-.21 [-.47, .08]	.20 [-.07, .40]
BAS Fun	.05 [-.23, .33]	-.01 [-.36, .28]	.09 [-.25, .36]	-.11 [-.37, .16]	.14 [-.16, .42]	.05 [-.23, .33]	.14 [-.15, .39]	.09 [-.19, .35]	.07 [-.25, .39]	.17 [-.14, .40]
BAS Reward Responsivity	.01 [-.26, .26]	-.10 [-.43, .18]	.02 [-.36, .33]	-.10 [-.40, .18]	.06 [-.26, .35]	.00 [-.36, .31]	-.09 [-.47, .22]	.08 [-.18, .34]	-.20 [-.44, .10]	.19 [-.10, .44]
BIS Total	.02 [-.26, .29]	-.17 [-.47, .13]	-.32 [-.59, .08]	-.20 [-.51, .09]	-.18 [-.46, .20]	-.18 [-.48, .14]	-.08 [-.30, .20]	-.02 [-.32, .28]	-.24 [-.51, .04]	-.08 [-.31, .18]
PANAS PA	-.08 [-.36, .18]	-.20 [-.44, .07]	.07 [-.24, .40]	-.11 [-.37, .20]	.14 [-.15, .42]	.20 [-.04, .43]	.04 [-.31, .43]	.01 [-.27, .35]	-.21 [-.48, .09]	.16 [-.18, .42]
PANAS NA	.09 [-.17, .31]	-.09 [-.31, .12]	-.04 [-.36, .34]	**-.29 [-.52, -.01]**	.08 [-.20, .37]	**.31 [.01, .59]**	.28 [-.06, .63]	-.15 [-.42, .14]	-.05 [-.34, .24]	-.03 [-.30, .23]
MASQ General Distress Anxious	.07 [-.23, .34]	.10 [-.20, .36]	-.07 [-.35, .25]	-.16 [-.39, .14]	-.06 [-.35, .24]	.20 [-.07, .49]	.25 [-.09, .55]	-.22 [-.47, .08]	.03 [-.25, .29]	-.14 [-.36, .09]
MASQ Anxious Arousal	.13 [-.14, .38]	.12 [-.12, .37]	-.06 [-.36, .31]	-.11 [-.37, .28]	-.11 [-.40, .19]	.28 [-.04, .61]	.28 [-.03, .65]	**-.29 [-.51, -.02]**	.06 [-.20, .33]	-.16 [-.36, .06]
MASQ General Distress Depressive	.05 [-.25, .31]	**.28 [.02, .60]**	.07 [-.26, .45]	-.06 [-.49, .27]	-.13 [-.43, .21]	**.33 [.03, .57]**	.30 [-.02, .54]	-.16 [-.43, .17]	.15 [-.23, .41]	-.15 [-.44, .21]
MASQ Anhedonic Depression	-.08 [-.39, .23]	.18 [-.15, .51]	.03 [-.29, .36]	.01 [-.33, .27]	-.05 [-.37, .27]	.03 [-.24, .31]	.10 [-.23, .38]	.11 [-.20, .38]	.14 [-.16, .38]	-.16 [-.42, .12]
SHAPS	-.09 [-.35, .20]	-.12 [-.35, .16]	.11 [-.21, .42]	-.10 [-.35, .16]	.27 [-.01, .51]	.03 [-.29, .41]	.01 [-.30, .42]	.21 [-.16, .49]	-.16 [-.43, .17]	.24 [-.04, .45]
PROMIS-D	-.04 [-.35, .25]	.26 [-.03, .53]	.16 [-.17, .48]	-.21 [-.60, .08]	.07 [-.25, .40]	.26 [-.07, .54]	.16 [-.12, .44]	.01 [-.28, .33]	.02 [-.38, .26]	-.09 [-.31, .23]

Model 4

ORL Estimates (Modeled Jointly across Sessions)

	Session 1					Session 2				
Self-Report Collected at Session 1	***A+***	***A-***	***K***	**β*f***	**β*p***	***A+***	***A-***	***K***	**β*f***	**β*p***
BAS Total	.00 [-.33, .31]	-.13 [-.48, .19]	.04 [-.28, .34]	-.12 [-.41, .19]	.10 [-.24, .40]	.01 [-.31, .32]	.00 [-.31, .31]	.08 [-.24, .38]	-.12 [-.40, .17]	.17 [-.13, .43]
BAS Drive	-.01 [-.36, .33]	-.22 [-.53, .12]	.01 [-.30, .31]	-.11 [-.41, .17]	.05 [-.28, .36]	.00 [-.34, .31]	-.05 [-.33, .25]	.07 [-.24, .36]	-.17 [-.44, .10]	.16 [-.13, .39]
BAS Fun	.07 [-.22, .36]	.03 [-.31, .32]	.09 [-.19, .35]	-.06 [-.33, .21]	.12 [-.17, .38]	.05 [-.22, .32]	.14 [-.16, .39]	.10 [-.17, .35]	.05 [-.25, .35]	.15 [-.14, .38]
BAS Reward Responsivity	-.05 [-.36, .24]	-.13 [-.43, .19]	.00 [-.33, .31]	-.13 [-.43, .15]	.09 [-.21, .37]	-.01 [-.36, .27]	-.08 [-.42, .22]	.04 [-.28, .33]	-.19 [-.43, .08]	.14 [-.15, .42]
BIS Total	-.07 [-.35, .23]	-.18 [-.46, .12]	-.28 [-.55, .09]	-.24 [-.53, .03]	-.16 [-.41, .18]	-.15 [-.46, .16]	-.06 [-.30, .22]	-.20 [-.47, .15]	-.26 [-.53, .01]	-.11 [-.34, .16]
PANAS PA	-.01 [-.27, .27]	-.15 [-.41, .13]	.07 [-.24, .38]	-.14 [-.40, .20]	.15 [-.14, .43]	.10 [-.12, .34]	-.03 [-.37, .33]	.10 [-.20, .41]	-.21 [-.46, .09]	.18 [-.14, .43]
PANAS NA	.24 [-.04, .52]	.05 [-.22, .29]	-.08 [-.37, .27]	-.28 [-.52, .01]	.05 [-.23, .31]	**.29 [.01, .59]**	.20 [-.09, .52]	-.07 [-.36, .27]	-.16 [-.42, .11]	.04 [-.25, .27]
MASQ General Distress Anxious	.20 [-.10, .46]	.20 [-.10, .44]	-.18 [-.43, .14]	-.12 [-.36, .18]	-.10 [-.36, .18]	.24 [-.02, .50]	.27 [-.05, .53]	-.17 [-.43, .14]	.01 [-.26, .27]	-.14 [-.37, .11]
MASQ Anxious Arousal	.28 [-.01, .55]	.22 [-.06, .44]	-.17 [-.43, .20]	-.08 [-.34, .31]	-.15 [-.40, .14]	**.31 [.01, .60]**	.27 [-.01, .57]	-.18 [-.44, .17]	.04 [-.22, .30]	-.17 [-.41, .09]
MASQ General Distress Depressive	.30 [-.02, .56]	**.40 [.17, .63]**	-.06 [-.37, .27]	.01 [-.45, .35]	-.15 [-.44, .19]	**.39 [.10, .60]**	**.36 [.09, .56]**	-.09 [-.35, .22]	.10 [-.32, .37]	-.17 [-.45, .20]
MASQ Anhedonic Depression	.00 [-.29, .29]	.21 [-.06, .50]	-.01 [-.31, .29]	.05 [-.31, .32]	-.10 [-.40, .21]	.05 [-.21, .32]	.13 [-.16, .40]	-.04 [-.33, .26]	.12 [-.20, .35]	-.18 [-.41, .10]
SHAPS	-.13 [-.41, .25]	-.11 [-.40, .18]	.17 [-.16, .46]	-.13 [-.38, .15]	**.30 [.02, .52]**	-.06 [-.37, .35]	-.04 [-.35, .34]	.22 [-.13, .54]	-.19 [-.42, .10]	**.31 [.02, .50]**
PROMIS-D	.16 [-.18, .45]	**.31 [.05, .54]**	.05 [-.24, .39]	-.17 [-.60, .11]	.00 [-.28, .34]	.26 [-.06, .53]	.21 [-.06, .46]	.01 [-.27, .32]	-.07 [-.48, .18]	-.07 [-.30, .28]

a At session 1, the n for MASQ and PROMIS-D correlations is 46; the n for all other session 1 correlations is 48

b At session 2, for all Model 1 correlations n is 44; for Model 2, the n for MASQ and PROMIS-D correlations is 46 and the n for all other correlations is 48

Supplemental Discussion

ORL model decomposes task behavior into distinct psychological processes

While the IGT summary score confounds different psychological mechanisms driving gross IGT performance (Ahn, et al., 2016; Almy, et al., 2018; Cauffman, et al., 2012; Lin, et al., 2007), the ORL (Haines, et al., 2018), decomposes performance into a theoretically rich set of process-level behavioral metrics, including Reward Learning Rate, Punishment Learning Rate, Win Frequency Sensitivity, Perseveration Tendency, and Memory Decay. Learning Rate, rather than referring to amount or quality of learning *across the whole task*, reflects the degree to which an individual "weights" prediction error for given outcomes on a trial-by-trial basis to update value estimations. Thus, higher Reward and Punishment Learning Rates can be thought of as more volatile trial-level updating in a reward or loss domain, respectively. Optimal learning rate depends on task structure (Bishop & Gagne, 2018; Nussenbaum & Hartley, 2019); thus, higher learning rates are not necessarily conducive to better performance. Indeed, we found better IGT performance was negatively associated with Reward Learning Rate but positively associated with Punishment Learning Rate. Another ORL parameter, Win Frequency Sensitivity, represents subjective value for reward frequency, rather than magnitude, and is a type of reward sensitivity commonly exhibited by healthy participants on the IGT (Haines, et al., 2018; Steingroever, et al., 2013). The ORL also provides metrics for Perseveration Tendency, or the consistency of a participant's choices irrespective of outcomes, and Memory Decay, an index of the participant's memory for which choices they selected in the past. Together, these five parameters allow a much more nuanced and fine-grained understanding of individual behavior on the IGT.

Potential model adaptations to address outstanding issues

One potential model adaptation might include examining potential change in learning parameters across engagement with the IGT during a single session (i.e., learning rate may change from the beginning to the end of the task). Another traditional scoring method for the IGT (not explored in the current paper) is examining change in deck selection across early versus later blocks in the game. Future modeling work might compare this alternative traditional scoring approach to a generative model that allows learning parameters to vary across the task.

Future research should also examine learning parameters longitudinally across multiple task administrations. In considering how to model data collected across multiple task administrations, it may prove useful to explicitly model potential carry-over learning effects as participants engage with the IGT multiple times. For example, it is possible that trial-and-error learning may be faster on a second task encounter, which could be modeled by assuming larger mean priors on learning rates for a second testing session. Alternatively, longitudinal estimation of learning parameters may be improved by assuming *different* priors on reward versus punishment learning rates, based on the parameter estimates from an individual's initial task performance.

Another possibility is that different types of learning (e.g., continuous trial-and-error versus one-shot or cateogry learning) may guide task performance once people learn the "rules" of the task. For example, the IGT has a valence structure such that two choices lead to long-term gains while two the other two choice lead to long-term losses. If participants learn this rule, they may develop an abstract representation of the task wherein their goal is to determine which category (good or bad) a deck belongs to, and then make a choice based on the learned category rather than information about expected value or win frequency. If this is the case, test-retest reliability would naturally be low. A potential solution could be to frame the IGT as a category learning task, wherein

the different monetary outcomes shown on each trial are used as stimulus features to categorize the deck as either "good" or "bad" (see Turner et al., 2018). Whether a deck is "good" or "bad" itself could then be determined with expected value information as in the ORL model. If reliance on trial-and-error versus category learning is then treated as a mixture model, it is possible that learning model parameters could themselves be consistent across time, and that changes in behavior across sessions could be due to an increased reliance (i.e. a higher mixture probability) on category learning. We anticipate that future work would benefit greatly by exploring these possibilities.

Another issue (raised above) was that some associations between behavioral estimates and self-report were inconsistent across testing sessions. It is important to reiterate here that two-step correlations between measures will be attenuated by any lack of precision in the individual measures (Spearman, 1904). As self-report measures were not incorporated into the current hierarchical model (and thus self-report measure uncertainty was not modified), associations between self-report and behavioral measures are attenuated in proportion to this lack of precision, which decreases power and could result in inconsistent associations. Future work should address this issue by incorporating self-report measures of theoretical importance directly into the hierarchical models (e.g., Kildahl, et al., 2020; Kvam, et al., 2021; Vandekerckhove, 2014), and/or including in the hierarchical model multiple self-report assessments across time along with modeling the covariance between these measures (as we have done here with the behavioral data). These modeling strategies would likely enhance the reliability of associations between behavior and self-report measures.

Supplemental References

Turner, B. M., Schley, D. R., Muller, C., & Tsetsos, K. (2018). Competing theories of multialternative, multiattribute preferential choice. *Psychological Review*, *125*(3), 329–362. https://doi.org/10.1037/rev0000089
